# p47^phox^ deficiency improves cognitive impairment and attenuates tau hyperphosphorylation in mouse models of AD

**DOI:** 10.1186/s13195-020-00714-2

**Published:** 2020-11-12

**Authors:** Ping Gong, Yan-qing Chen, Ai-hua Lin, Hai-bo Zhang, Yan Zhang, Richard D. Ye, Yang Yu

**Affiliations:** 1 Engineering Research Center of Cell and Therapeutic Antibody, Ministry of Education, and School of Pharmacy, Shanghai Jiao Tong University, Shanghai, 200240 China; 2 Kobilka Institute of Innovative Drug Discovery, School of Life and Health Sciences, The Chinese University of Hong Kong, Shenzhen, 518172 China

**Keywords:** Alzheimer’s disease, Nicotinamide adenine dinucleotide phosphate oxidase, p47^phox^, *Ncf1*, NOX2, Cognition, Tau, Neurons, Astrocytes

## Abstract

**Background:**

Alzheimer’s disease (AD) is characterized by progressive memory loss and cognitive impairment. The aggregation of amyloid β (Aβ) and hyperphosphorylated tau protein are two major pathological features of AD. Nicotinamide adenine dinucleotide phosphate oxidase (NADPH oxidase, NOX) has been indicated in Aβ pathology; however, whether and how it affects tau pathology are not yet clear.

**Methods:**

The role of NOX2 in cognitive function, amyloid plaque formation, and tau hyperphosphorylation were examined in *APP/PS1* transgenic mice mated with p47^phox^-deficient mice (with deletion of the gene of neutrophil cytosolic factor 1, *Ncf1*) and/or in p47^phox^-deficient mice receiving intracerebroventricular (ICV) injection of streptozotocin (STZ). The cognitive and non-cognitive functions in these mice were assessed by Morris water maze, Rotarod test, open field, and elevated plus maze. Aβ levels, amyloid plaques, p47^phox^ expression, and astrocyte activation were evaluated using immunofluorescence staining, ELISA, and/or Western blotting. Cultured primary neuronal cells were treated with okadaic acid or conditioned media (CM) from high glucose-stimulated primary astrocytes. The alteration in tau pathology was determined using Western blotting and immunofluorescence staining.

**Results:**

Deletion of the gene coding for p47^phox^, the organizer subunit of NOX2, significantly attenuated cognitive impairment and tau pathology in these mice. p47^phox^ deficiency decreased the activation of astrocytes but had no effect on Aβ levels and amyloid plaque formation in the brains of aged *APP/PS1* mice, which displayed markedly increased expression of p47^phox^ in neurons and astrocytes. Cell culture studies found that neuronal p47^phox^ deletion attenuated okadaic acid-induced tau hyperphosphorylation at specific sites in primary cultures of neurons. CM from high glucose-treated WT astrocytes increased tau hyperphosphorylation in primary neurons, whereas this effect was absent from p47^phox^-deficient astrocytes.

**Conclusions:**

These results suggest that p47^phox^ is associated with cognitive function and tau pathology in AD. p47^phox^ expressed in neurons contributes to tau hyperphosphorylation directly, while p47^phox^ in astrocytes affect tau hyperphosphorylation by activating astrocytes indirectly. Our results provide new insights into the role of NOX2 in AD and indicate that targeted inhibition of p47^phox^ may be a new strategy for the treatment of AD.

## Background

Alzheimer’s disease (AD) is a chronic neurodegenerative disease characterized by progressive memory loss and cognitive function deficits [1]. The accumulation of extracellular senile plaques composed of aggregated amyloid β (Aβ) and intracellular neurofibrillary tangles (NFTs) consisting of hyperphosphorylated tau are the two major pathological hallmarks of AD [1, 2]. At present, there is no cure for AD or treatment to delay its progress. The most widely accepted pathogenic hypothesis of AD is the amyloid cascade hypothesis, proposing that Aβ accumulation is a major early event in the pathogenesis of AD [3, 4]. However, during the last decade, no clinical trials with Aβ-targeted drugs have been successful [5], suggesting that treatment for subsequent events of AD, such as oxidative stress, neuroinflammation, and tau hyperphosphorylation may be equally important.

Oxidative stress, which can be induced by excessively generated reactive oxygen species (ROS), has been indicated to contribute to Aβ pathology and AD pathogenesis [6]. Endogenous ROS in the brain is mainly produced by the nicotinamide adenine dinucleotide phosphate (NADPH) oxidase (NOX), an oxidant-producing enzyme first described in neutrophils [7, 8]. In mammals, the NOX family includes NOX1, NOX2, NOX3, NOX4, NOX5, DUOX1, and DUOX2 isoforms [9]. The active NOX complex identified in phagocytes is composed of the cytosolic subunits p47^phox^, p67^phox^, p40^phox^, the small GTPase *Rac*, and the membrane subunits gp91^phox^ (NOX2) and p22^phox^. Upon activation, the phosphorylated p47^phox^ initiates the formation of a cytosolic complex, and then facilitates their translocation to the plasma membrane for the assembly of an activated NOX2 complex [9]. Of these subunits, p47^phox^ plays a key role as the organizer of phagocyte oxidase subunits for NOX2 activation [9]. Translocation of p47^phox^ and p67^phox^ from the cytosol to the membrane and the upregulation of NOX1 and NOX3 genes have been found in the brain of AD patients, suggesting that the NOX proteins may play an important role in AD [10, 11]. NOX2 is expressed in neurons, astrocytes, microglia, and the cerebral vascular cells in the brain [12]. Studies have found that Aβ plaques or peptides can upregulate and activate NOX2 in astrocytes and microglia, leading to ROS production and neuronal cell death [13, 14]. The activation of NOX2 in neurons also induces neuronal death [15]. In addition, high levels of NOX2 have been found in the cerebral arteries [16]. Several evidences indicate that NOX2 is involved in the mechanism of cerebrovascular damage induced by Aβ accumulation in AD mouse models [17–19]. All these results indicate the involvement of NOX2 in Aβ pathology of AD; however, how NOX2 affects the tau pathology and whether NOX2 participates in tau pathology through different cell types are not yet clear.

In this study, we used the *Ncf1* (coding for p47^phox^) knockout mice to explore the role of NOX2 in tau pathology. We have established two mouse models of AD, one is the *APP/PS1* double transgenic mice mated with the *Ncf1*^*−/−*^ mice, and the other involves intracerebroventricular (ICV) injection of streptozotocin (STZ) into WT and *Ncf1*^*−/−*^ mice. The results have shown that p47^phox^ deficiency significantly improves cognitive impairment and attenuates tau hyperphosphorylation in the brain of these AD model mice. p47^phox^ deficiency has no effect on the content and the accumulation of Aβ in *APP/PS1* mice. The expression of p47^phox^ in neurons and astrocytes are markedly increased in *APP/PS1* mice. Moreover, p47^phox^ deficiency is related to the decreased activation of astrocytes in these mice. Finally, primary cell culture studies showed that p47^phox^ in neurons and astrocytes is involved in tau pathology.

## Materials and methods

### Reagents and antibodies

Dulbecco’s modified Eagle’s medium (DMEM), neurobasal-A, B-27^®^, and trypsin-ethylenediaminetetraacetic acid (trypsin-EDTA) were purchased from Gibco (Invitrogen, Carlsbad, CA). The BCA protein assay kit, normal goat serum (NGS), 4,6-diamidino-2-phenylindole (DAPI), and phenylmethanesulfonyl fluoride (PMSF) were obtained from Beyotime Institute of Biotechnology (Nantong, Jiangsu, China). Okadaic acid (OA) and glucose were purchased from Sangon Biotech (Shanghai) Co., Ltd. (Shanghai, China). Mouse anti-total tau (Tau5) and phospho-tau (AT8) antibodies and rabbit anti-phospho-tau pSer199, pThr205, pSer396, and pSer404 antibodies were obtained from Invitrogen (Carlsbad, CA). Mouse anti-non-phospho-tau (Tau1) and anti-glial fibrillary acidic protein (GFAP) antibodies were obtained from Merck (Darmstadt, Germany). Rabbit anti-β-Amyloid and mouse anti-β-Amyloid antibodies were purchased from Cell Signaling Technology (Danvers, MA) and Biolegend (San Diego, CA), respectively. Rabbit anti-MAP2 and mouse anti-GFAP Cy3™ antibodies were from Sigma-Aldrich (St. Louis, MO). Mouse anti-p47^phox^ antibody was purchased from Santa Cruz Biotechnology (Dallas, Texas). Rabbit antibodies against p67^phox^, NOX2/gp91^phox^, and amyloid precursor protein (APP) were obtained from Abcam (Cambridge, MA). Rabbit anti-Iba1 antibody was obtained from FUJIFILM Wako Pure Chemical (Osaka, Japan). Mouse anti-glyceraldehyde-3-phosphate dehydrogenase (GAPDH) and rabbit anti-β-Actin antibodies were from Biosynthesis Biotechnology Co., Ltd. (Beijing, China). Alexa Fluor 488-conjugated anti-rabbit IgG, Alexa Fluor 488-conjugated anti-mouse IgG, Alexa Fluor 568-conjugated anti-rabbit IgG, and Alexa Fluor 568-conjugated anti-mouse IgG secondary antibodies were from Invitrogen. IRDye® 800CW and IMDye® 800CW secondary antibodies were from LI-COR, Inc. (Lincoln, NE). Other reagents were purchased from Sigma-Aldrich (St. Louis, MO).

### Animals and treatments

The p47^phox^-deficient (*Ncf1* knock out, *Ncf1*^−/−^) mice in C57BL/6 J background were kindly provided by Dr. Steven M Holland (National Institutes of Health, Bethesda, MD), as reported previously [20]. Mouse genotypes were determined by PCR. The following primers were used: *Ncf1* F: 5′-ACA TCA CAG GCC CCA TCA TCC TCC-3′; *Ncf1* R: 5′-GGA GAG CCC CCT TTC TCT CCC TCA-3′; *Ncf1* neo: 5′-CAA CGT CGA GCA CAG CTG CGC AAG-3′. The expected size of the PCR product using *Ncf1* F and R primers is 650 bp, and the product using *Ncf1* F and neo is 900 bp. The mutant mice were identified by the presence of a 900 bp PCR product, and the WT mice were identified by the presence of a 650 bp PCR product. Heterozygous mice contained both of the products. *APP/PS1* transgenic mice in C57BL/6 J background (*APP*_*SWE*_/*PS1ΔE9*^+/−^, stock number 005864) were obtained from the Jackson Laboratory (Bar Harbor, ME). p47^phox^-deficient mice were crossed to *APP/PS1* mice to generate *APP/PS1*-*Ncf1*^+/−^ mice, and then the latter were further crossed with *Ncf1*^+/−^ mice to create the following four groups: WT (*APP/PS1*^−/−^-*Ncf1*^+/+^), *APP/PS1* (*APP/PS1*^+/−^-*Ncf1*^+/+^), *Ncf1*^−/−^ (*APP/PS1*^−/−^-*Ncf1*^−/−^), and *APP/PS1*-*Ncf1*^−/−^ (*APP/PS1*^+/−^-*Ncf1*^−/−^). Mouse genotypes were determined by PCR. The mice of different genotypes were kept in separate cages. Mice of the same genotype were housed (3–5 mice per cage) with a 12/12 h light/dark cycle with ad libitum access to food and water. The housing, breeding, and animal experiments were in accordance with the National Institutes of Health Guide for the Care and Use of Laboratory Animals, with procedures approved by the Biological Research Ethics Committee, Shanghai Jiao Tong University. All four groups of male and female mice with 9 months of age (WT, 6 males and 4 females; *APP/PS1*, 4 males and 4 females; *Ncf1*^−/−^, 5 males and 5 females; *APP/PS1*-*Ncf1*^−/−^, 5 males and 5 females) and 12 months of age (WT, 10 males and 10 females; *APP/PS1*, 10 males and 1 female; *Ncf1*^−/−^, 5 males and 4 females; *APP/PS1*-*Ncf1*^−/−^, 4 males and 3 females) were subjected to a battery of behavioral tests. The behavioral tests were performed by two independent investigators blinded to the genotype of the mice.

**Fig. 1:**
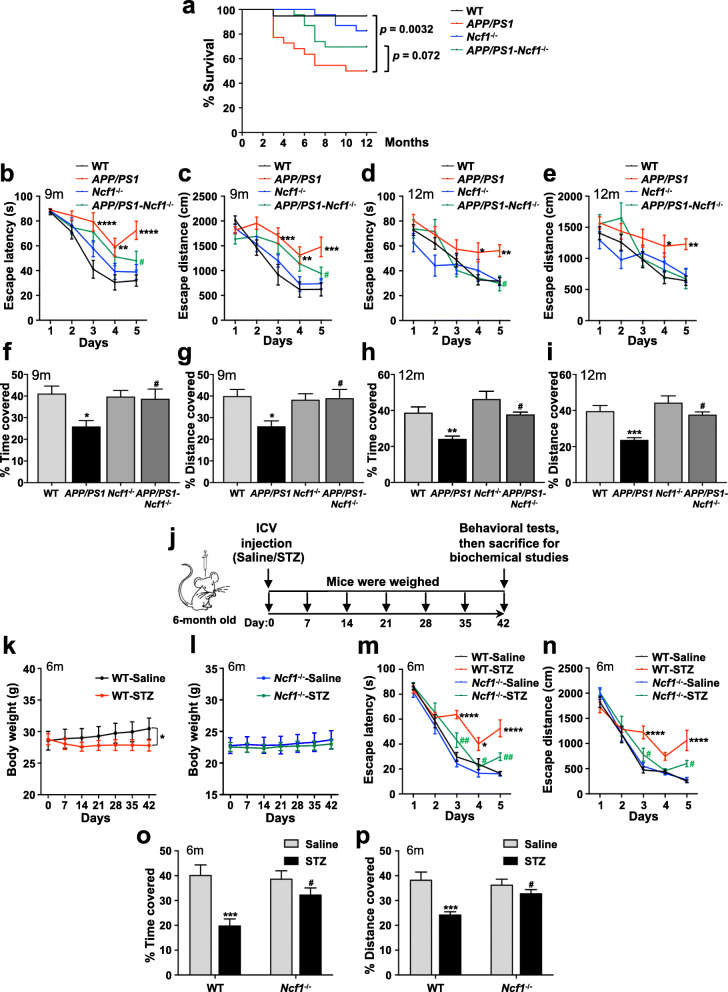
p47^phox^ deficiency improves cognitive impairment in AD mice. **a** Survival curves for WT, *APP/PS1*, *Ncf1*^−/−^, and *APP/PS1*-*Ncf1*^−/−^ mice. Both male and female mice were used. Data are mean ± SEM, with 19–23 mice in each group. Spatial reference learning and memory were assessed in Morris water maze (MWM). Mice aged 9 and 12 months were trained in MWM for consecutive 5 days. The time spent (**b**, **d**) and the distance traveled (**c**, **e**) to reach the escape platform are shown. The probe trials were tested 24 h after the last day of training. The percentage of time spent (**f**, **h**) and distance traveled (**g**, **i**) within the target quadrant are shown. Two-way ANOVA: **b**, interaction *F* (12, 170) = 1.697 *p* = 0.0712, days *F* (4, 170) = 39.10 *p* < 0.0001, genotype *F* (3, 170) = 14.51 *p* < 0.0001; **c**, interaction *F* (12, 170) = 2.117 *p* = 0.0182, days *F* (4, 170) = 30.93 *p* < 0.0001, genotype *F* (3, 170) = 11.92 *p* < 0.0001; **d**, interaction *F* (12, 214) = 1.098 *p* = 0.3632, days *F* (4, 214) = 20.56 *p* < 0.0001, genotype *F* (3, 214) = 8.071 *p* < 0.0001; **e**, interaction *F* (12, 214) = 1.221 *p* = 0.2699, days *F* (4, 214) = 14.47 *p* < 0.0001, genotype *F* (3, 214) = 7.906 *p* < 0.0001. one-way ANOVA: **f**, *F* (3, 34) = 3.511 *p* = 0.0255; **g**, *F* (3, 34) = 3.773 *p* = 0.0194; **h**, *F* (3, 43) = 6.889 *p* = 0.0007; **i**, *F* (3, 43) = 7.541 *p* = 0.0004. Both male and female mice were used. Data are mean ± SEM, with 7–20 mice in each group. **j** Schematic representation of study design. Mice aged 6 months received intracerebroventricular (ICV) injection of either saline or 3.0 mg/kg STZ on day 0. Mice were weighed once every 7 days until day 42; all mice were subjected to MWM. The bodyweight of WT (**k**) and *Ncf1*^−/−^ mice (**l**) receiving STZ or saline was recorded. MWM tests were performed. The time spent (**m**) and the distance traveled (**n**) to reach the platform are shown. For the probe trial, the percentage of time spent (**o**) and distance traveled (**p**) within the target quadrant are shown. Two-way ANOVA: **k**, interaction *F* (6, 84) = 0.2830 *p* = 0.9435, days *F* (6, 84) = 0.1030 *p* = 0.9959, treatment *F* (1, 84) = 4.767 *p* = 0.0318; **l**, interaction *F* (6, 84) = 0.009671 *p* > 0.9999, days *F* (6, 84) = 0.1032 *p* = 0.9959, treatment *F* (1, 84) = 0.4472 *p* = 0.5055; **m**, interaction *F* (12, 120) = 3.961 *p* < 0.0001, days *F* (4, 120) = 131.2 *p* < 0.0001, treatment *F* (3, 120) = 25.31 *p* < 0.0001; **n**, interaction *F* (12, 120) = 3.722 *p* < 0.0001, days *F* (4, 120) = 118.8 *p* < 0.0001, treatment *F* (3, 120) = 12.80 *p* < 0.0001. One-way ANOVA: **o**, *F* (3, 24) = 9.267 *p* = 0.0003; **p**, *F* (3, 24) = 8.693 *p* = 0.0004. Both male and female mice were used. Data are mean ± SEM, with 6–8 mice in each group. *p < 0.05, **p < 0.01, ***p < 0.001, ****p < 0.0001 compared with WT or WT ICV-Saline mice. ^#^*p* < 0.05, ^##^*p* < 0.01 compared with *APP/PS1* or WT ICV-STZ mice

The ICV-STZ mice were established by giving a single dose of stereotaxic injection of STZ [2-deoxy-2-(3-(methyl-3-nitrosoureido)-D-glucopyranose)] into both lateral ventricles of the mice brain [21, 22]. WT and *Ncf1*^−/−^ mice aged 6 months were randomly divided into control and ICV-STZ groups respectively. After anesthetized by intraperitoneal injection of 5 mg/ml pentobarbital sodium, the mice were restrained onto a stereotaxic apparatus. Each mouse received a single ICV injection of 3.0 mg/kg STZ, which was freshly prepared in 0.9% saline, into both lateral brain ventricles. The bregma coordinates were − 0.3 mm posterior, ± 1.0 mm lateral, and − 2.5 mm below. As controls, ICV-saline mice were injected with an equal volume of sterile normal saline. All mice were placed on heating pads (37 °C) until recovered from surgery. The bodyweight of the mice was measured once every week. Six weeks after ICV injection, all mice (WT ICV-Saline, 5 males and 2 females; WT ICV-STZ, 4 males and 3 females; *Ncf1*^−/−^ ICV-Saline, 3 males and 5 females; *Ncf1*^−/−^ ICV-STZ, 1 male and 5 females) were subjected to a battery of behavioral tests (Fig. 1j). The behavioral tests were performed by two independent investigators blinded to the genotype of the mice.

### Morris water maze

Spatial reference learning and memory were evaluated in a water maze [23]. The test was performed in a white pool of 150 cm in diameter filled with water tinted with non-toxic white paint and maintained at room temperature. During training, an 8-cm diameter platform was submerged 1 cm below the water surface. All mice were given four trials per day for five consecutive days. The starting position was randomized among four quadrants of the pool every day. For each trial, the animal was given 90 s to locate the hidden platform. If a mouse failed to find the platform within 90 s, it was gently guided to it. At the end of each trial, the mouse was left on the platform for 20 s, then dried and returned to its home cage until the next trial. The probe trial was given 24 h after the last day of training. During probe trial, mice were allowed to swim in the pool without the platform for 60 s. The latency to reach the platform site (s) and swim distance (cm) were recorded using an automated tracking system (Smart video tracking system, Shanghai Mobile Datum, Shanghai, China).

### Accelerating Rotarod test

Motor coordination and balance of mice were tested by using a Rotarod test. Test on an accelerating Rotarod was conducted by three trials on a rotating cylinder. The speed increased steadily from 5 to 30 rpm over a 90-s period. The latency to fall off the Rotarod was recorded. Inter-trial intervals were 10–15 min for each mouse.

### Open field

Anxiety and exploratory activities were detected by an open field test. The testing apparatus was an open field (a polyvinyl chloride square arena, 80 × 80 cm, with walls 30 cm high), surmounted by a video camera connected to a computer. The arena was lit from the ceiling of the room with incandescent light, with a measurement inside the apparatus of 60 lx. Each mouse was placed individually in the corner and allowed to freely explore for 15 min. The 40-cm-diameter square defined the inner zone. The distance traveled in the arena, and the time and distance traveled in the center area (inner zone) were recorded and analyzed by Smart video tracking system (Shanghai Mobile Datum).

### Elevated plus maze

Elevated plus maze was used to test anxiety/emotionality of the mice. It consisted of four arms (30 × 5 cm) connected by a common 5 × 5 cm center area. Two opposite facing arms were open (open arms), whereas the other two facing arms were enclosed by 20-cm high walls (closed arms). The entire plus maze was elevated on a pedestal to a height of 82 cm above floor level in a separate room from the investigator. The mouse was placed onto the central area facing an open arm and allowed to freely explore the maze for a single 8-min session. Between each session, feces were cleared from the maze, and the maze floor was cleaned with 75% alcohol to remove any urine or scent cues. The time spent in open arms and closed arms were recorded by Smart video tracking system (Shanghai Mobile Datum).

### Tissue preparation

After behavioral tests, all mice were sacrificed by decapitation and their brains removed immediately. The hippocampi and cerebral cortices of the left hemisphere of the brain were dissected, flash frozen in dry ice, and stored at − 80 °C for biochemical analyses later. The right hemispheres of the brain were fixed with 4% paraformaldehyde in 0.1 M phosphate-buffered (PBS) for 24 h, followed by cryoprotection in 30% sucrose. Sagittal sections of 30 μm thickness were cut using a freezing sliding microtome. The sections were stored in glycol anti-freeze solution (ethylene glycol, glycerol, and 0.1 M PBS in 3:3:4 ratio) at − 20 °C until immunofluorescence staining. In this study, we included 3–6 mice/group for biochemical analyses and immunofluorescence staining.

### Western blot

Mouse brain tissue was homogenized in lysis buffer containing 50 mM Tris-HCL (pH 7.4), 100 mM NaF, 2 mM EDTA, 10 mM β-mercaptoethanol, 2 mM NaVanadate, 8.5% sucrose, 5 μg/ml aprotinin, 100 μg/ml leupeptin, and 5 μg/ml pepstatin. Protein concentrations were determined by using BCA Kits according to the manufacturer’s protocol. Quantitative homogenates were added 5 × sodium dodecyl sulfate (SDS) to heat for 10 min at 99 °C and resolved in 10% SDS-PAGE for separation. After separated, samples were transferred onto nitrocellulose membranes (GE Healthcare, Wauwatosa, WI), and the membranes were blocked with 5% non-fat milk for 1 h at room temperature. Then, the membranes were incubated with primary antibodies including Tau5 (1:1000), Tau1 (1:1000), the anti-phospho-tau antibodies pS199 (1:1000), pT205 (1:1000), pS396 (1:1000), pS404 (1:1000), anti-β-Amyloid (6E10, 1:2000), anti-APP (1:20000), anti-p47^phox^ (1:250), anti-p67^phox^ (1:1000), anti-NOX2/gp91^phox^ (1:1000), anti-GFAP (1:1000), anti-GAPDH (1:20000), and anti-β-Actin (1:20000), followed by the respective IRDye® 800CW or IMDye® 800CW secondary antibodies. The membranes were scanned using an Odyssey P140-CLx Infrared Imaging System (LI-COR, Inc.). Densitometric quantification of protein bands was analyzed using the ImageJ software (National Institutes of Health, Bethesda, MD).

### Immunofluorescence staining

Free-floating sections were processed for standard immunofluorescence staining [24]. Briefly, the sections were washed three times for 5 min with pre-cold 0.05 M Tris-buffered saline (TBS, containing 0.05 M Tris buffer and 9 g/L NaCl, pH 7.4), followed by blocked with 5% NGS in 0.05 M TBS (0.1% Tween-20) for 1 h at room temperature. Then, the sections were incubated with anti-β-amyloid antibody (1:200), anti-AT8 antibody (1:500), or anti-Iba1 antibody (1:500) in TBS at 4 °C overnight. After rinsing with TBS, the sections were further incubated with Alexa Fluor 488-conjugated anti-rabbit or Alexa Fluor 568-conjugated anti-mouse (1:500) secondary antibodies for 1 h at room temperature. After three washes in TBS, the sections were stained for nuclei with 100 ng/ml DAPI for 10 min and mounted on glass slides.

For double immunofluorescence staining of AT8 and thioflavin-S (Thio-S), the staining of AT8 was performed as described above. Then, the sections were rinsed in TBS and stained with 1% Thio-S for 10 min at room temperature. The sections were washed with 50% ethanol for two times. After three washes in TBS, the sections were stained for nuclei with 100 ng/ml DAPI for 10 min and mounted on glass slides. For double immunofluorescence staining of p47^phox^ and MAP2, the sections were first incubated with anti-p47^phox^ antibody (1:200) in TBS at 4 °C overnight and treated in Alexa Fluor 488-conjugated anti-mouse secondary antibody (1:500) for 1 h. The sections were rinsed in TBS, stained with anti-MAP2 antibody (1:200) at 4 °C overnight, and incubated with Alexa Fluor 568-conjugated anti-rabbit secondary antibody (1:500) for 1 h. For double immunofluorescence staining of p47^phox^ and GFAP, the sections were first incubated with anti-p47^phox^ antibody (1:200) in TBS at 4 °C overnight and incubated with Alexa Fluor 488-conjugated anti-mouse secondary antibody (1:500) for 1 h. Then, the sections were rinsed in TBS, stained with anti-GFAP-Cy3™ antibody (1:500) for 1 h. After double immunofluorescence staining, the sections were washed in TBS, stained for nuclei with 100 ng/ml DAPI for 10 min, and mounted on glass slides.

The fluorescent confocal images were taken on a laser-scanning confocal fluorescent microscope (TCS SP8, Leica Microsystems, Wetzlar, Germany). For quantification of the expression of p47^phox^, Aβ deposits, phosphorylated tau (AT8), astrocytes (GFAP), and microglia (Iba1), the relative immunofluorescence intensity was quantified using the ImageJ software. For quantification of colocalization of p47^phox^ with MAP2 or GFAP, the number of colocalized cells per square millimeter of the sections was calculated. The results were expressed as the means ± SEM based on 3–5 mice each group, or a minimum of four viewing fields for each region, using at least two mice.

### Aβ ELISA

Mouse brain tissue was homogenized in lysis buffer containing 50 mM Tris-HCL (pH 7.4), 100 mM NaF, 2 mM EDTA, 10 mM β-mercaptoethanol, 2 mM sodium vanadate, 8.5% sucrose, 5 μg/ml aprotinin, 100 μg/ml leupeptin, and 5 μg/ml pepstatin. The homogenate was centrifuged at 14000 rpm for 15 min at 4 °C. The supernatant (soluble fraction) was collected. The pellets were dissolved in guanidine HCl solution (6 M guanidine hydrochloride, 50 mM Tris-HCl, 1% PMSF, pH 8.0), cooled for 20 min in an ice bath, and centrifuged at 14000 rpm for 15 min at 4 °C. The supernatant (insoluble fraction) was collected. Protein concentrations were determined by using BCA Kits according to the manufacturer’s protocol. Then, soluble and insoluble Aβ_1–40_ and Aβ_1–42_ were measured using commercially available ELISA kits from Shanghai Westang Bio-tech Co., LTD (Shanghai, China), according to the manufacturer’s instructions.

### Primary neuronal cultures

Neuronal cultures were prepared in cortices obtained from 1-day-old WT and *Ncf1*^−/−^ mice as described before [25]. In brief, cerebral cortices were removed from the brains of mice, the meanings and microvessels were removed, and tissues were minced with a sterile razor blade. Tissues were digested with 0.025% trypsin-EDTA at 37 °C for 10 min. The cell suspension was filtered through a 200-mesh sieve, and cells were plated on poly-D-lysine-coated 12-well plates at a density of 10 × 10^5^ cells per well. Four hours later, the DMEM medium (containing 10% FBS, 100 U/ml of penicillin, and 100 μg/ml streptomycin sulfate) were replaced with neurobasal medium containing 2% B-27® supplements for 2 days. Culture medium was changed to neurobasal with 10% FBS and 5 μg/ml cytosine-β-D-arabinofuranoside (Ara-C) in the following 1 days, and then again switched back to neurobasal medium containing 2% B-27^®^ supplements. Experiments were performed on days 7–8 after initiation of the culture.

### Primary astrocyte culture

Astrocyte cultures were prepared from 1-day-old WT and *Ncf1*^−/−^ mice as described before [26]. Specifically, separated cells were cultured in poly-D-lysine-coated 75 cm^2^ flasks with DMEM medium (containing 10% FBS, 100 U/ml penicillin, and 100 μg/ml streptomycin sulfate). The medium was replenished on day 1 and day 3. On day 7, microglia cells in the culture flasks were shaken off at 260 × rpm for 2.5 h. Then, the remaining astrocytes were maintained in DMEM with 10% FBS until seeding into 24-well plates. Experiments were performed after one passage of the cells.

### Treatment with conditioned medium of astrocytes

When astrocytes cultured in plates grew to 70–80% confluence, the culture medium was replaced with fresh DMEM without FBS, or with the above medium containing 75 mM or 150 mM glucose. The cells were incubated for another 24 h and 48 h. At the end of the incubation, conditioned medium (CM) was collected, and cells were lysed in 5 × SDS buffer for immunoblotting analysis. The astrocyte CM collected at 24 h or 48 h was added to the culture of neurons from WT mice for another 48 h. The neuronal cell lysate was prepared and analyzed by SDS-PAGE and Western blotting as detailed above.

### Statistical analyses

All data are presented as the means ± SEM. Mean values of paired groups were analyzed using Student’s *t* test. Mean of multiple groups were analyzed using one-way analysis of variance (ANOVA), followed by Tukey’s post hoc test. The survival rate of the mice was analyzed using Gehan-Breslow-Wilcoxon test. The bodyweight of the mice, Morris water maze, and Rotarod test were analyzed using two-way ANOVA. All analyses were performed with the statistical software GraphPad Prism 8 (San Diego, CA), and *p* value less than 0.05 was considered statistically significant.

## Results

### Genetic deficiency of p47^phox^ improves cognitive impairment in AD mice

To assess the potential role of p47^phox^ in AD, we generated the *APP/PS1* transgenic and p47^phox^-deficient (*Ncf1* knockout, *APP/PS1*-*Ncf1*^−/−^) mice. We crossed *APP/PS1* transgenic mice on a C57BL/6 background to the *Ncf1*^−/−^ mice (also on a C57BL/6 background) to generate *APP/PS1*-*Ncf1*^+/−^ mice, then crossed *APP/PS1*-*Ncf1*^+/−^ mice with *Ncf1*^−/−^ mice to generate *APP/PS1*-*Ncf1*^−/−^ mice. We first observed the mortality in these mice. All four groups of mice (WT, *APP/PS1*, *Ncf1*^−/−^, and *APP/PS1*-*Ncf1*^−/−^ mice) initially showed healthy. However, at the age of 3 months, there was a significant increase in the mortality rate of *APP/PS1* mice compared with the other three groups of mice (Fig. 1a). The mortality rate of *APP/PS1*-*Ncf1*^−/−^ mice did not increase until the age of 5 months. By the age of 12 months, 30% of *APP/PS1*-*Ncf1*^−/−^ mice died, compared with 55% of *APP/PS1* mice (*p* = 0.072, Fig. 1a). These data show that p47^phox^ deficiency decreases the mortality in *APP/PS1* mice.

Next, we investigated the effect of p47^phox^ on cognitive performance. We tested all four groups of mice aged 9 and 12 months in Morris water maze (MWM). The result showed that *APP/PS1* mice at both ages took a longer time (Fig. 1b, d) and swam a longer distance (Fig. 1c, e) than the WT mice to find the escape platform, indicating a learning impairment in *APP/PS1* mice. However, in *APP/PS1*-*Ncf1*^−/−^ mice, a shorter escape latency and swimming distance on day 5 to reach the escape platform were observed compared with the *APP/PS1* mice (Fig. 1b–e), suggesting that p47^phox^ deficiency improves learning of the *APP/PS1* mice. After one day off, probe trials were performed on day 6 to evaluate the spatial reference memory of the mice. As expected, *APP/PS1* mice aged 9 months and 12 months spent less time and traveled less distance in the target quadrant than WT mice, indicating an impairment of spatial reference memory (Fig. 1f–i). However, p47^phox^ deficiency increased the time spent and distance swam of *APP/PS1*-*Ncf1*^−/−^ mice in the target quadrant compared with *APP/PS1* mice (Fig. 1f–i). All these findings suggest that p47^phox^ deficiency improves the spatial learning and memory of *APP/PS1* mice.

Furthermore, we established another mouse model of AD to confirm the effect of p47^phox^ on cognitive function. The ICV injection of STZ mouse is widely recognized as a mouse model of sporadic AD [22]. STZ is a diabetogenic compound. In ICV-STZ mice, brain insulin resistance, impaired glucose metabolism, aggregation of tau, and learning and memory deficits have been reported [27, 28]. The ICV-STZ AD model was generated by ICV injection of STZ into *Ncf1*^−/−^ mice and WT littermates, using normal saline as a control (ICV-Saline) (Fig. 1j). As STZ-induced animal models are characterized by insulin deficiency accompanied by lower body weight, changes in the body weight after ICV injection were monitored. ICV-STZ mice had lower body weight compared with ICV-Saline mice (Fig. 1k), consistent with previous reports [29]. However, in the *Ncf1*^−/−^ group, ICV-STZ mice had similar body weight with ICV-Saline mice (Fig. 1l), suggesting that p47^phox^ deficiency attenuated the decrease in body weight of ICV-STZ mice. For the MWM test, the ICV-STZ mice took more time and swam a longer distance than ICV-saline mice to find the escape platform on day 5 (Fig. 1m, n). This result is consistent with what was reported previously [30]. In the *Ncf1*^−/−^ group, no difference was observed between the STZ and saline groups, and both of these groups showed less time spent and shorter distance swum to the escape platform on day 5, compared with the WT ICV-STZ group. Furthermore, probe trails test showed that p47^phox^ deficiency increased the time spent and distance swum in the target quadrant of mice receiving ICV-STZ, compared with WT ICV-STZ mice (Fig. 1o, p). Consistent with the results of *APP/PS1* mouse model, all these findings suggest that p47^phox^ deficiency improves cognitive impairment in AD.

In addition to the cognitive functions, general behaviors were also evaluated in *APP/PS1* mice. Motor coordination and balance ability were measured using Rotarod test. *APP/PS1* mice aged 9 months and 12 months displayed a decreasing trend in fall latency compared with WT mice (Additional file 1: Fig. S1a and g). p47^phox^ deficiency did not change the fall latency of *APP/PS1* mice (Additional file 1: Fig. S1a and g), indicating that p47^phox^ has no effect on the motor coordination and balance of AD mice. Then, we evaluated spontaneous exploratory activity of mice. We observed that no significant difference in the total distance explored between four groups in the open field test (Additional file 1: Fig. S1b and h), suggesting that p47^phox^ has no effect on spontaneous exploratory ability in mice. Next, we measured anxiety in mice using open field and elevated plus maze tests. We observed that *APP/PS1* mice spent less time in the center area of the open field (Additional file 1: Fig. S1c, e, i, and k) and in the open arms of the elevated plus maze (Additional file 1: Fig. S1d, f, j, and l), compared with WT mice. Interestingly, similar to *APP/PS1* mice, the p47^phox^-deficient mice also showed shorter time staying in the center area and in the open arms in these two tests compared with WT mice (Additional file 1: Fig. S1c-f, i-l). However, p47^phox^ deficiency did not seem to affect the time *APP/PS1* mice spent in the center area and in the open arms (Additional file 1: Fig. S1c-f, i-l). These results suggest that although p47^phox^ deficiency induces anxiety in mice, it does not aggravate the anxiety in *APP/PS1* mice.

### p47^phox^ deficiency does not affect Aβ levels in *APP/PS1* mice

**Fig. 2:**
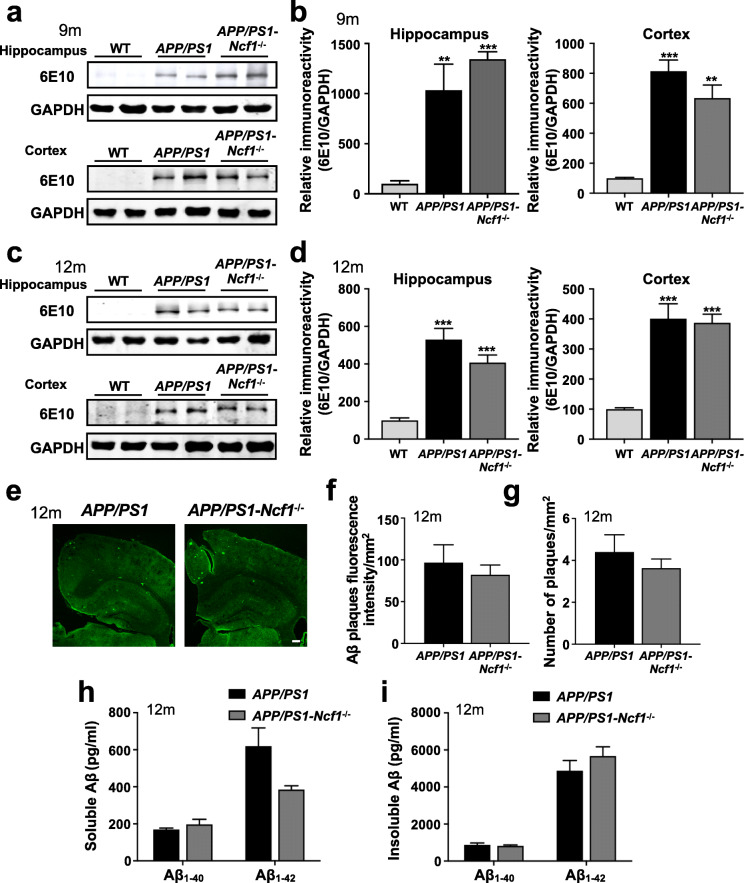
p47^phox^ deficiency does not affect Aβ levels in *APP/PS1* mice. Representative Western blots showing Aβ deposits in the hippocampus and the cortex of WT, *APP/PS1*, and *APP/PS1*-*Ncf1*^−/−^ mice aged 9 months (**a**) and 12 months (**c**). **b**, **d** Quantification of immunoreactivity of Western blots, normalized against GAPDH. One-way ANOVA: **b**, hippocampus *F* (2, 8) = 27.46 *p* = 0.0003, cortex *F* (2, 6) = 31.56 *p* = 0.0007; **d**, hippocampus *F* (2, 8) = 33.30 *p* = 0.0001, cortex *F* (2, 9) = 26.55 *p* = 0.0002. Both male and female mice were used. Data are mean ± SEM, with 4–5 mice in each group. **p < 0.01, ***p < 0.001 compared with WT mice. **e** Representative immunofluorescence images of Aβ deposits in the brain of *APP/PS1* and *APP/PS1*-*Ncf1*^−/−^ mice, aged 12 months. Scale bar, 500 μm. Quantification of the fluorescence intensity (**f**) and number (**g**) of Aβ deposits are shown. Both male and female mice were used. Data are mean ± SEM, with 4–5 mice in each group. **h**, **i** Soluble and insoluble Aβ_1–40_ and Aβ_1–42_ from the brain of 12-month-old mice were measured using ELISA. Both male and female mice were used. Data shown are the means ± SEM, with 4 mice in each group

In order to verify whether the improvement of cognitive deficits is related to the change of Aβ accumulation in the brain of *APP/PS1*-*Ncf1*^−/−^ mice, we analyzed the brain Aβ in the *APP/PS1* and *APP/PS1*-*Ncf1*^−/−^ mice used in MWM tests. As shown in Fig. 2a–d, Aβ levels in the hippocampus and the cortex were significantly increased in 9- and 12-month-old *APP/PS1* mice compared with age-matched WT mice. However, p47^phox^ deficiency did not affect Aβ levels in the brain of *APP/PS1* mice. In addition to western blotting, immunofluorescence staining was performed to determine Aβ deposits of mice at the age of 12 months. Similarly, no significant differences were observed in the number of Aβ plaques and the area occupied by Aβ (fluorescence intensity) in the brains of *APP/PS1* mice and *APP/PS1*-*Ncf1*^−/−^ mice (Fig. 2e–g), consistent with the results of western blotting. Furthermore, ELISA was performed to determine soluble and insoluble Aβ in the brain of mice. Soluble and insoluble Aβ_1–40_ and Aβ_1–42_ levels did not differ between *APP/PS1* mice and *APP/PS1*-*Ncf1*^−/−^ mice (Fig. 2h, i). Although soluble Aβ_1–42_ showed a trend of decrease in *APP/PS1*-*Ncf1*^−/−^ mice compared with *APP/PS1* mice, the difference was not statistically significant (Fig. 2h). These results suggest that p47^phox^ may not affect the content and accumulation of Aβ in vivo. Aβ is derived by proteolytic processing from the APP; therefore, we further verify whether the deletion of p47^phox^ affects APP expression. As expected, a marked increase in APP expression was found in the brain of *APP/PS1* mice compared with the WT mice (Additional file 1: Fig. S2a-d). However, no significant change was observed in *APP/PS1*-*Ncf1*^−/−^ mice compared with the *APP/PS1* mice (Additional file 1: Fig. S2a-d), suggesting the p47^phox^ deficiency has no effect on the expression of APP in AD mice.

### p47^phox^ deficiency attenuates tau hyperphosphorylation in AD mouse models

**Fig. 3:**
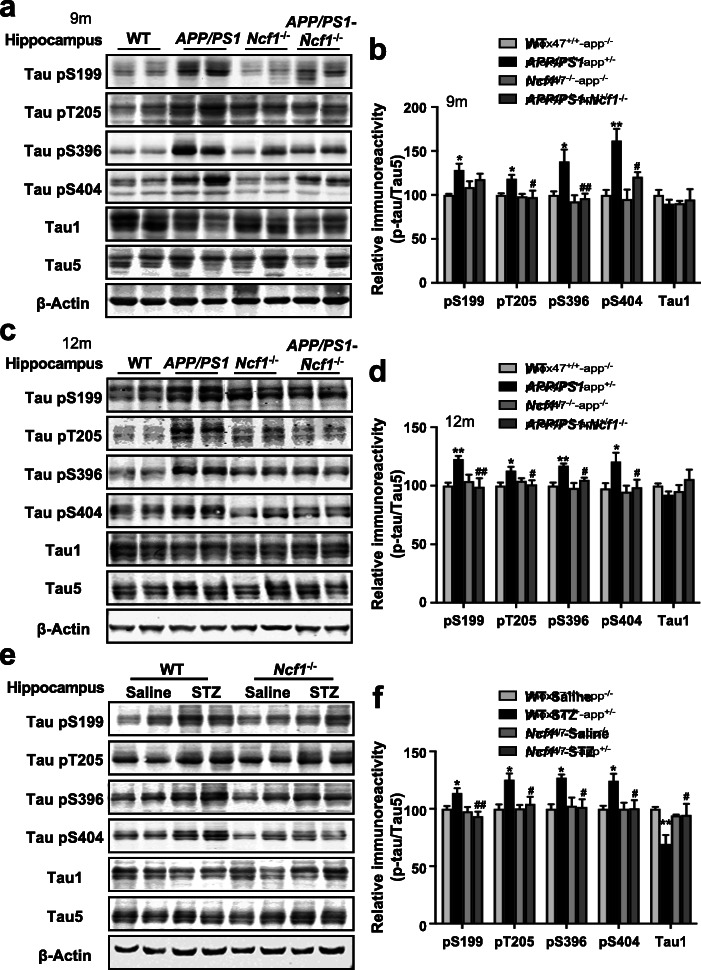
p47^phox^ deficiency attenuates tau hyperphosphorylation in the hippocampus of *APP/PS1* mice and ICV-STZ mice. Representative Western blots showing tau phosphorylation at S199, T205, S396, and S404 in the hippocampus of WT, *APP/PS1*, *Ncf1*^−/−^, and *APP/PS1*-*Ncf1*^−/−^ mice aged 9 months (**a**) and 12 months (**c**). The levels of non-phosphorylated tau (Tau1) and total tau (Tau5) were also measured. **b**, **d** Quantification of the immunoreactivity of Western blots, normalized against total tau. **e** Representative Western blots showing tau phosphorylation in the hippocampus of WT and *Ncf1*^−/−^ mice receiving ICV injection of STZ or saline at the age of 6 months. **f** Quantification of the immunoreactivity of Western blots, normalized against total tau. One-way ANOVA: **b**, pS199 *F* (3, 13) = 4.165 *p* = 0.0285, pT205 *F* (3, 16) = 4.079 *p* = 0.0250, pS396 *F* (3, 16) = 6.366 *p* = 0.0048, pS404 *F* (3, 14) = 9.922 *p* = 0.0009, Tau1 *F* (3, 11) = 0.3631 *p* = 0.7810; **d**, pS199 *F* (3, 31) = 5.512 *p* = 0.0037, pT205 *F* (3, 25) = 3.722 *p* = 0.0243, pS396 *F* (3, 23) = 7.629 *p* = 0.0010, p-S404 *F* (3, 28) = 3.995 *p* = 0.0174, Tau1 *F* (3, 31) = 1.013 *p* = 0.4000; **e**, pS199 *F* (3, 20) = 5.508 *p* = 0.0064, pT205 *F* (3, 16) = 5.331 *p* = 0.0097, pS396 *F* (3, 19) = 3.539 *p* = 0.0344, pS404 *F* (3, 20) = 4.595 *p* = 0.0133, Tau1 *F* (3, 20) = 4.383 *p* = 0.0159. Both male and female mice were used. Data are mean ± SEM, with 4–6 mice in each group. *p < 0.05, **p < 0.01 compared with WT or WT ICV-Saline mice; ^#^*p* < 0.05, ^##^*p* < 0.01 compared with *APP/PS1* or WT ICV-STZ mice

The above results showed that p47^phox^ deficiency reduced the mortality and improved the learning and memory impairment of AD mice; however, it has no effect on the accumulation of Aβ in the brain of AD mice. Accumulating evidence demonstrates that the cognitive impairment of AD is closely related to the pathology of tau [31, 32]. Therefore, we further analyzed the phosphorylation of tau protein in the brain of the mice used in MWM tests. We examined the levels of total tau (Tau5 antibody), non-phosphorylated tau (Tau1 antibody), and phosphorylated tau at several AD-related amino acid sites including Ser199, Thr205, Ser396, and Ser404 in the hippocampus and the cortex of mice. As expected, a marked increase was observed in tau phosphorylation at Ser199, Thr205, Ser396, and Ser404 in the hippocampus (Fig. 3a-d) as well as in the cortex (Additional file 1: Fig. S3a-d) of *APP/PS1* mice aged 9 months and 12 months, compared with age-matched WT mice. Correspondingly, a decrease was also displayed in the level of non-phosphorylated tau in *APP/PS1* mice (Fig. 3a–d; Additional file 1: Fig. S3a-d). However, no significant difference was observed in tau phosphorylation at these sites, nor was the non-phosphorylated tau different between *Ncf1*^−/−^ mice and *APP/PS1*-*Ncf1*^−/−^ mice (Fig. 3a–d; Additional file 1: Fig. S3a-d). These results indicate that p47^phox^ deficiency attenuates the hyperphosphorylation of tau in *APP/PS1* mice. In addition to Western blot, immunofluorescence staining was performed to confirm the effect of p47^phox^ on tau pathology. Frozen slices were stained with AT8, which were wildly used for the detection of tau pathology, such as aggregated tau, neuropil threads (NTs), and NFTs [33–35] . Significant tau aggregation and NTs were observed in CA1, CA3, and dentate gyrus (DG) regions of the hippocampus and the cortex of *APP/PS1* mice aged 12 months (Additional file 1: Fig. S4a, b). However, p47^phox^ deficiency attenuated tau aggregation and NTs in *APP/PS1* mice (Additional file 1: Fig. S4a, b). Moreover, we also detected NP tau, another major type of AD-related tau pathology, which aggregates in dystrophic neurites surrounding Aβ plaques [36]. NP tau was examined by AT8 and thioflavin-S (Thio-S). As shown in Additional file 1: Fig. S5, p47^phox^ deficiency reduced the number of NP tau in *APP/PS1*-*Ncf1*^−/−^ mice compared with *APP/PS1* mice. All these data suggest that p47^phox^ deficiency attenuates tau pathology in *APP/PS1* mice.

Then, we also analyzed tau phosphorylation in the brain of ICV-STZ injection mice. Similar to *APP/PS1* mice, in the hippocampus and the cortex of ICV-STZ mice, tau phosphorylation at Ser199, Thr205, Ser396, and Ser404 was increased and non-phosphorylated tau was decreased when compared with ICV-saline mice (Fig. 3e, f; Additional file 1: Fig. S3e and f). In the *Ncf1*^−/−^ mice, however, no significant difference was found in the phosphorylated and non-phosphorylated tau in the brain of ICV-STZ mice compared with ICV-saline mice (Fig. 3e, f; Additional file 1: Fig. S3e and f). These results confirmed that p47^phox^ is involved in tau pathology in AD.

**Fig. 4:**
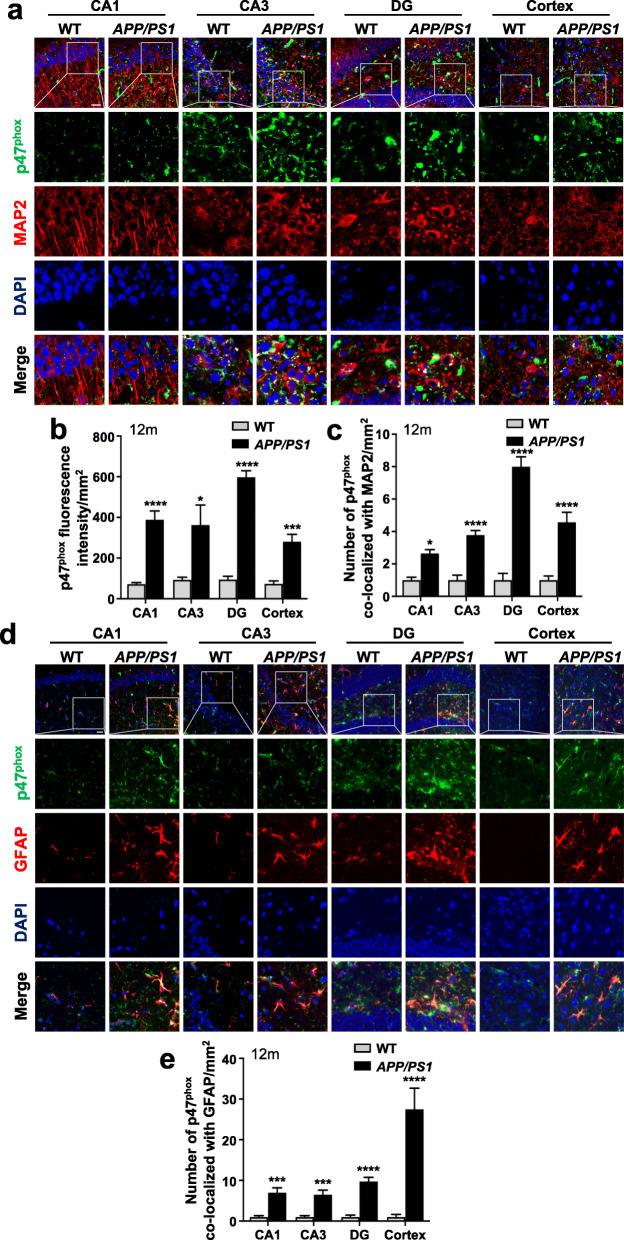
Upregulation of p47^phox^ in neurons and astrocytes of aged *APP/PS1* mice brain. **a** Serial sections of WT and *APP/PS1* mice aged 12 months were stained for p47^phox^ (*green fluorescence*) and MAP 2 (*red fluorescence*) as described in the “Materials and methods” section. Nuclei were stained with DAPI (*blue fluorescence*). The scale bar in the upper left panel is 25 μm. Selected areas are enlarged by five times and shown as combined as well as individual fluorescence stains. Quantification of the fluorescence of p47^phox^ (**b**) and the number of p47^phox^ colocalized with MAP 2 (**c**) are shown. **d** Serial sections of WT and *APP/PS1* mice aged 12 months were stained for p47^phox^ (*green fluorescence*) and GFAP (*red fluorescence*) as described in the “Materials and methods” section. Nuclei were stained with DAPI (*blue fluorescence*). The scale bar in the upper left panel is 25 μm. Selected areas are enlarged by five times and shown as combined as well as individual fluorescence stains. **e** Quantification of the number of p47^phox^ colocalized with GFAP is shown. Both male and female mice were used. Data are mean ± SEM, based on four viewing fields for each region, using at least two mice. *p < 0.05, ***p < 0.001, ****p < 0.0001 compared with WT mice

To verify the mechanism by which NOX affects tau phosphorylation, we first examined the expression of the three main subunits of NOX, including p47^phox^, p67^phox^, and NOX2/gp91^phox^, in the brain of *APP/PS1* mice. Western blotting showed that the expression level of these three subunits, especially that of p47^phox^, was significantly upregulated in the brain of *APP/PS1* mice aged 9 months and 12 months when compared with age-matched WT mice (Additional file 1: Fig. S6a-d). In addition to Western blotting, immunofluorescence staining was performed to confirm the expression and distribution of p47^phox^ in the brain. Frozen brain slices were stained with primary anti-p47^phox^ antibody and then treated with corresponding secondary antibody (green fluorescence). As shown in Fig. 4a, b, there was a significant upregulation of p47^phox^ in CA1, CA3, and DG regions of the hippocampus as well as in the cortex of *APP/PS1* mice aged 12 months, compared with age-matched WT mice. To identify the cellular origin of p47^phox^, double immunostaining was conducted for p47^phox^ (green fluorescence) and the cell-specific markers MAP2 (neurons, red fluorescence) and GFAP (astrocytes, red fluorescence). p47^phox^ was colocalized with neurons (Fig. 4c) and astrocytes (Fig. 4d, e) in the hippocampus and the cortex of mice aged 12 months. Moreover, the number of p47^phox^ colocalized with neurons and astrocytes was significantly increased in the *APP/PS1* mice, compared with the WT mice (Fig. 4c, e). Increased expression of p47^phox^ on the membrane and in the cytoplasm was also observed in both types of cells in the *APP/PS1* mice (Fig. 4a, d).

### p47^phox^ expressed in neurons affects tau phosphorylation

**Fig. 5:**
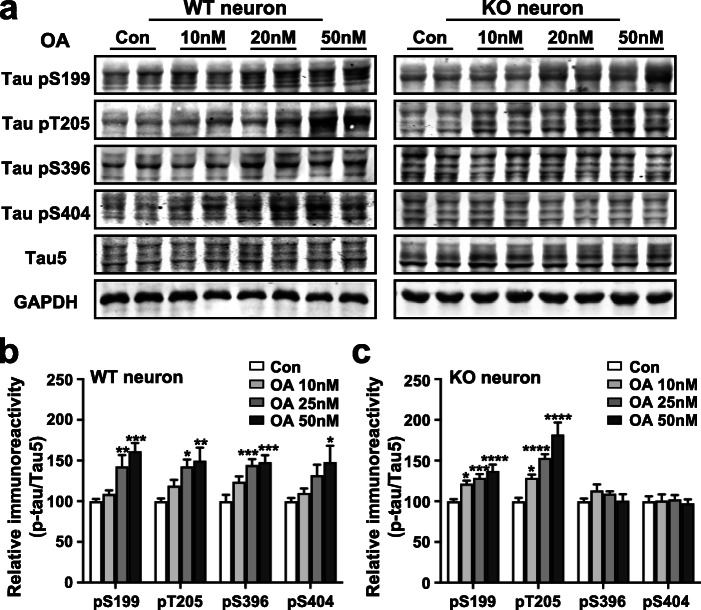
Neuronal p47^phox^ deficiency alleviates tau hyperphosphorylation at specific sites in neurons induced by OA treatment. Freshly isolated neurons from WT and *Ncf1*^−/−^ mice were exposed to OA (10 nM, 25 nM, or 50 nM) for 12 h. **a** Representative Western blots showing tau hyperphosphorylation at S199, T205, S396, and S404 in primary neurons. **b**, **c** Quantification of the immunoreactivity of Western blots, normalized against total tau (Tau5). One-way ANOVA: **b**, pS199 *F* (3, 32) = 10.16 *p* < 0.0001, pT205 *F* (3, 32) = 5.317 *p* = 0.0044, pS396 *F* (3, 32) = 8.480 *p* = 0.0003, pS404 *F* (3, 28) = 3.743 *p* = 0.0223; **c**, pS199 *F* (3, 31) = 10.26 *p* < 0.0001, pT205 *F* (3, 30) = 22.43 *p* < 0.0001, pS396 *F* (3, 31) = 1.171 *p* = 0.3366, pS404 *F* (3, 31) = 0.1162 *p* = 0.9499. Data are mean ± SEM from three separate experiments, each in duplicate or triplicate. *p < 0.05, **p < 0.01, ***p < 0.001, ****p < 0.0001 compared with medium without OA treatment

Next, we examined whether p47^phox^ expressed in neurons and astrocytes is involved in the regulation of tau phosphorylation. It was shown in Fig. 4a that increased expression of p47^phox^ was found in neurons. Therefore, we propose that neuronal p47^phox^ regulates tau phosphorylation in neurons. We have found that p47^phox^ deficiency attenuates the hyperphosphorylation of tau in *APP/PS1* mice and ICV injection of STZ mice. Thus, a cellular model of AD with okadaic acid (OA) treatment was used to induce tau hyperphosphorylation in primary cultures of neurons from WT or *Ncf1*^−/−^ newborn mice and to verify whether p47^phox^ deletion could alleviate tau hyperphosphorylation. As expected, OA treatment induced a significant concentration-dependent increase of tau phosphorylation at Ser199, Thr205, Ser396, and Ser404 sites in primary cultures of neurons from WT mice (Fig. 5a, b). However, for primary neurons of *Ncf1*^−/−^ mice, OA treatment could not induce tau hyperphosphorylation at Ser396 and Ser404, but tau phosphorylation at Ser199 and Thr205 was still increased (Fig. 5a, c). These results indicate that the neuronal p47^phox^ participates in OA-induced tau hyperphosphorylation at specific sites.

### p47^phox^ expressed in astrocytes affects tau phosphorylation

Since the neuronal p47^phox^ is involved in tau phosphorylation directly, we further investigate whether and how p47^phox^ expressed in astrocytes participates in regulating tau phosphorylation in neurons indirectly. We have shown that both activated astrocytes and p47^phox^ expressed in astrocytes were significantly increased in the brain of *APP/PS1* mice (Fig. 4d, e). Then, we verified whether p47^phox^ deletion affected the activation of astrocytes in these mice. As shown in Additional file 1: Fig. S7a and b, the expression of GFAP was significantly increased in CA and DG areas of the hippocampus as well as in the cortex of *APP/PS1* mice at the age of 9 and 12 months, compared with age-matched WT mice. However, p47^phox^ deficiency obviously inhibited the activation of astrocytes in *APP/PS1* mice (Additional file 1: Fig. S7a and b). These results suggest that p47^phox^ participates in the activation of astrocytes in AD mice. In addition, studies have found that p47^phox^ is highly expressed in microglia and NADPH oxidase plays a vital role in microglia activation [37]. Thus, we detected the effect of p47^phox^ on microglia activation in *APP/PS1* mice. We found that compared with the WT mice, microglia showed a certain degree of activation in the hippocampus and the cortex of 12-month-old *APP/PS1* mice (Additional file 1: Fig. S8). p47^phox^ deficiency inhibited the activation of microglia in *APP/PS1* mice (Additional file 1: Fig. S8), suggesting that p47^phox^ is involved in microglia activation in AD mice. However, the activation of astrocytes in aged *APP/PS1* mice and the inhibition of p47^phox^ deletion on astrocyte activation seem to be more obvious than microglia. Therefore, we further examined whether p47^phox^ expressed in astrocytes could affect tau phosphorylation by activating astrocytes.

**Fig. 6:**
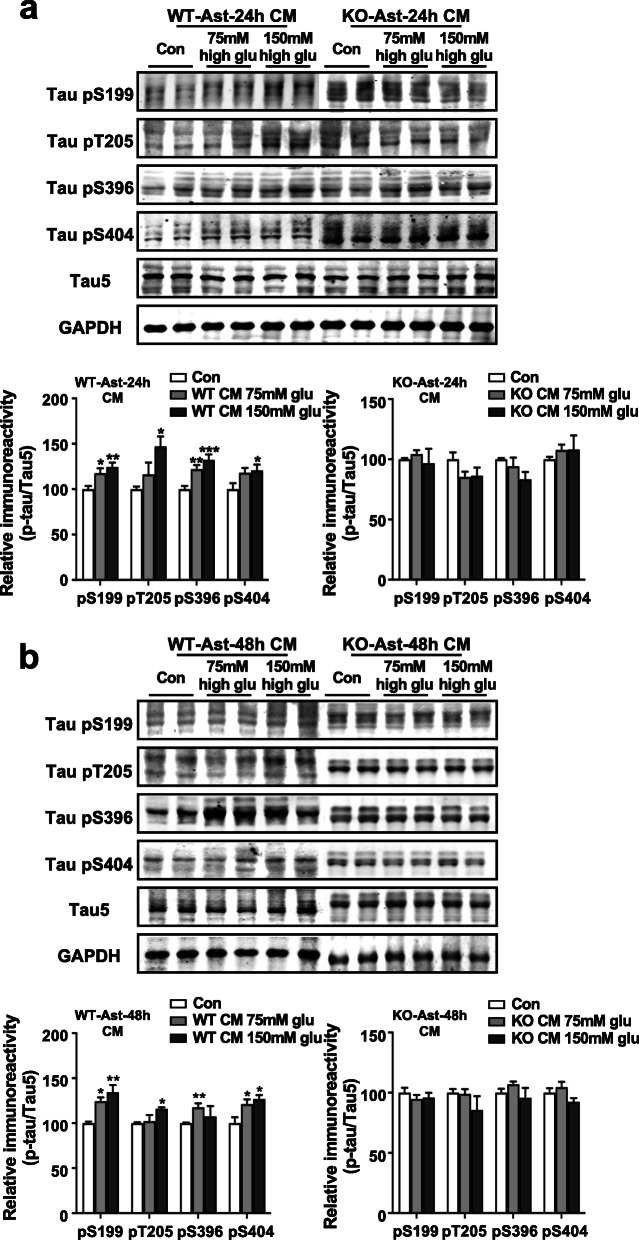
p47^phox^ in astrocytes affects tau phosphorylation by activating astrocytes. Freshly isolated neurons from WT mice were cultured for 48 h with conditioned medium (CM) of WT and *Ncf1*^−/−^ astrocytes that were exposed to high glucose (75 mM and 150 mM) or normal medium for 24 h (**a**) and 48 h (**b**). Representative Western blots showing tau hyperphosphorylation at S199, T205, S396, and S404 in primary neurons. The levels of total tau (Tau5) were also measured. Quantification of the immunoreactivity of Western blots, normalized against total tau (Tau5). One-way ANOVA: **a**, WT-Ast, pS199 *F* (2, 32) = 7.126 *p* = 0.0028, pT205 *F* (2, 8) = 5.410 *p* = 0.0327, pS396 *F* (2, 19) = 11.07 *p* = 0.0006, pS404 *F* (2, 21) = 3.466 *p* = 0.0500; KO-Ast, pS199 *F* (2, 9) = 0.2762 *p* = 0.7649, pT205 *F* (2, 9) = 2.046 *p* = 0.1851, pS396 *F* (2, 9) = 2.323 *p* = 0.1537, pS404 *F* (2, 9) = 0.3956 *p* = 0.6844; b, WT-Ast, pS199 *F* (2, 9) = 10.95 *p* = 0.0039, pT205 *F* (2, 8) = 7.484 *p* = 0.0147, pS396 *F* (2, 8) = 14.10 *p* = 0.0024, pS404 *F* (2, 8) = 6.255 *p* = 0.0231; KO-Ast, pS199 *F* (2, 16) = 0.5406 *p* = 0.5927, pT205 *F* (2, 16) = 1.599 *p* = 0.2327, pS396 *F* (2, 16) = 1.380 *p* = 0.2800, pS404 *F* (2, 16) = 2.149 *p* = 0.1490. Data are mean ± SEM from three separate experiments, each in duplicate or triplicate. *p < 0.05, **p < 0.01, ***p < 0.001 compared with medium without high glucose

Impaired cerebral glucose metabolism is one of the pathological features of AD [38]. Our previous study has found that high glucose treatment (75 mM and 150 mM) has no effect on tau phosphorylation and the viability of cultured primary neurons, but could induce the activation of astrocytes [26]. Here, we first tested whether p47^phox^ could affect high glucose activation of primary cultures of astrocytes. As shown in Additional file 1: Fig. S9a and b, high glucose (75 mM and 150 mM) induced a significant increase of GFAP expression in WT astrocytes after 24 and 48 h of treatment. However, this effect was absent in *Ncf1*^−/−^ astrocytes (Additional file 1: Fig. S9a and c), suggesting that p47^phox^ is involved in high glucose-induced activation of astrocytes. To further investigate how p47^phox^ in astrocytes participates in regulating tau phosphorylation in neurons, primary neurons from WT mice were cultured in the conditioned media (CM) collected from WT and *Ncf1*^−/−^ astrocytes after exposure to high glucose. We found that primary neurons cultured with CM from WT astrocytes displayed increased tau phosphorylation at Ser199, Thr205, Ser396, and Ser404 sites; however, primary neurons cultured with CM from *Ncf1*^−/−^ astrocytes did not (Fig. 6). These results suggest that p47^phox^ in astrocytes also plays a key role in the upregulation of tau phosphorylation, which involves mediators from the activated astrocytes.

## Discussion

The NADPH oxidase NOX2 subunit has been found to participate in Aβ pathology, suggesting that NOX2 may play a role in AD pathogenesis [39]. However, whether and how it affects the tau pathology, one of the major neuropathological characteristics of AD, are not quite clear. Accumulating evidence indicates that the cognitive impairment of AD is more closely related to tau pathology than other factors [40, 41]. In the present study, we demonstrated that p47^phox^, the organizer subunit of NOX2, plays a key role in cognitive function, tau hyperphosphorylation, and astrocyte activation in AD mouse models used in this study. Furthermore, in vitro primary cell culture experiments confirmed that p47^phox^ expressed in neurons contributes to tau hyperphosphorylation directly, whereas p47^phox^ in astrocytes affects tau hyperphosphorylation indirectly by activating astrocytes. Our results provide new insights into the role of p47^phox^-dependent NOX2 in AD.

In this study, we evaluated the effect of p47^phox^ on cognition and tau phosphorylation using two mouse models of AD, the *APP/PS1* double transgenic mice and ICV injection of STZ mice. These two mouse models are the well-known and widely used experimental models of familial AD and sporadic AD, respectively [22, 42–45]. We have found that p47^phox^ deficiency significantly attenuates cognitive deficits and tau hyperphosphorylation in these mouse models. Furthermore, p47^phox^ deletion seems to reduce the mortality of *APP/PS1* mice and does not affect the content and accumulation of Aβ in these mice. Park et al. reported that deletion of the gene coding for the catalytic subunit NOX2/gp91^phox^ does not develop oxidative stress, cerebrovascular dysfunction, or behavioral deficits in aged Tg2576 mice, indicating the contribution of cerebrovascular damage induced by NOX2 to the behavioral impairment in AD mice [39]. Interestingly, our results and theirs have found that the improvement in the cognition of AD mice lacking p47^phox^ or NOX2/gp91^phox^ does not accompany the reduction in brain Aβ levels or amyloid plaques. Our recent studies also found that deletion of the chemokine-like receptor 1 (CMKLR1) or formyl peptide receptor 2 (FPR2), the Aβ clearance receptors, improved the cognitive deficits of *APP/PS1* mice and/or ICV-STZ mice and attenuated tau hyperphosphorylation in the brain of these mice [30, 46]. Therefore, all these observations suggest that although Aβ accumulation is an early event in the pathogenesis of AD, the subsequent events such as NOX2-derived oxidative stress and tau hyperphosphorylation are more relevant to the cognitive impairment in advanced AD.

In addition to the cognitive function tests, we examined the non-cognitive behaviors of these aged AD mice. We have observed that p47^phox^ deficiency has no effect on motor coordination/balance and spontaneous exploratory activity of the WT mice or *APP/PS1* mice aged 9 and 12 months. However, compared with the WT mice, an increased anxiety of p47^phox^-deficient mice was observed, and p47^phox^ deficiency dose not aggravate the anxiety of *APP/PS1* mice. This result suggests that the p47^phox^ may participate in the anxiety of aged mice under physiological conditions, but it does not affect the anxiety of mice under AD pathological conditions. Also, Kishida et al have found that NOX2/gp91^phox^ deficiency resulting from deletion of mouse *Cybb* does not result in generalized anxiety or depression in mice at the age of 2–3 months [47]. Another study reported that *Cybb* deletion ameliorates chronic mild stress (CMS)-induced anxiety-like behaviors, but not depression-like behaviors in the young mice [48]. All these data indicate that inhibition of p47^phox^ may induce anxiety in aged mice, but it has no effect on the young mice and aged AD mice. Therefore, this may do not affect the potential of p47^phox^ inhibitors as therapeutic drugs for AD.

We have observed increased expression of p47^phox^ in the membrane and cytoplasm of neurons and astrocytes in aged *APP/PS1* mouse brain, compared with that of WT mice. Additionally, p47^phox^ deletion reduces GFAP expression in vivo and in vitro, suggesting involvement of p47^phox^-dependent NOX2 in the activation of astrocytes. Therefore, we postulate that p47^phox^ in neurons regulates tau phosphorylation directly, while p47^phox^ in astrocytes regulates tau phosphorylation indirectly by activating astrocytes. We also detected the effect of p47^phox^ on microglia activation. We found that microglia are activated in *APP/PS1* mice to a certain extent, and p47^phox^ deficiency inhibits the activation of microglia. Studies have shown that p47^phox^ is highly expressed in microglia and NADPH oxidase plays a key role in microglia activation [37]. The activation of microglia is related to the pathology of tau [24, 49–52]. Therefore, p47^phox^ may affect the cognition and tau pathology of AD mice via regulating the activation of microglia. However, astrocytes, the most abundant neuroglial cells in the brain, are also activated in AD [53]. DaRocha-Souto et al. reported that the number of reactive astrocytes is highly correlated with memory impairment and neuronal cell loss in AD mice [54]. Also, several studies indicate the interplays between reactive astrocytes and tau pathology [26, 49, 50, 55]. Our results show that p47^phox^ deficiency alleviates astrocyte activation, and p47^phox^ expressed in astrocytes is involved in tau hyperphosphorylation. All these data suggest that p47^phox^ participates in tau pathology by regulating glia cell activation.

Our results have shown that the neuronal p47^phox^ participates in the regulation of OA-induced tau hyperphosphorylation at Ser396 and Ser404 sites. OA, an inhibitor of protein phosphatase 1 (PP1) and 2A (PP2A), is widely used in cellular models to induce AD-like pathologies, including the hyperphosphorylation of tau [56]. It has been reported that PP2A is involved in AT2R (angiotensin II type-2 receptor)-induced PKC inhibition, thereby preventing NOX2 activation and ROS production [57]. Furthermore, NOX2 inhibition or p47^phox^ deletion prevents lipopolysaccharide (LPS) and interferon-gamma (IFN-γ)-induced activation of PP2A [58]. These findings indicate the interaction between PP2A and NOX2, but whether PP2A participates in the regulation of p47^phox^ in neurons on tau hyperphosphorylation needs further investigation. In addition, our results from the transfer of astrocyte-conditioned medium to primary cultures of neurons indicate that p47^phox^ in astrocytes is involved in activating astrocytes and inducing their release of regulator(s), which leads to tau hyperphosphorylation in neurons. Several evidences have shown that p47^phox^-dependent NOX2 activation induces ROS production in cultured astrocytes and/or brain slices [59–62]. In addition to ROS, it remains possible that other mediator(s) induced by activation of NOX2 in astrocytes may participate in tau phosphorylation in neurons. Altogether, our findings indicate that p47^phox^-dependent NOX2 is a potential target for AD therapy, and selective inhibition of p47^phox^ may provide a new strategy for the treatment of AD.

### Limitations

This study has several limitations. First, by the age of 12 months, *APP/PS1*-*Ncf1*^−/−^ mice displayed a decreasing trend in mortality rate compared with *APP/PS1* mice, whereas there was no significant difference in statistical analysis (*p* = 0.072). The current sample size of mice is a bit small. In addition, whether there is a causal link between the decrease of tau hyperphosphorylation and the improvement in cognitive function observed in *APP/PS1* mice lacking p47^phox^ has not been verified. Finally, we used mice of two sexes for experiments. We did not find that gender has a significant effect on the results of this study. Except for 12-month-old mice, the bodyweight of *APP/PS1* male mice is slightly higher than that of *APP/PS1*-*Ncf1*^−/−^ male mice, while there is no difference in the bodyweight of female mice.

## Conclusions

The NADPH oxidase NOX2 subunit has been found to participate in Aβ pathology, indicating that it may play a role in AD pathogenesis. Using two mouse models of AD, we demonstrate in this study that p47^phox^, the organizer subunit of NOX2, is associated with cognitive function and tau hyperphosphorylation in neurons. Neuronal p47^phox^ affects tau phosphorylation directly, whereas p47^phox^ in astrocytes contributes to tau phosphorylation indirectly by activating astrocytes. Our findings support the notion that p47^phox^ is a potential target for AD therapy.

## Supplementary Information

**Additional file 1: Figure S1.** The effect of p47^phox^ deficiency on the locomotor, spontaneous exploratory, and anxiety of *APP/PS1* mice. The locomotion and spontaneous exploratory activity of mice aged 9 (a, b) and 12 (g, h) months was evaluated on Rotarod and open field tests, respectively. The anxiety of mice aged 9 months (c, d) and 12 months (i, j) was examined in open field and an elevated plus maze. Representative traces of a mouse’s movements during the open field test (e, k) and the elevated plus maze (f, l) are shown. **Figure S2.** p47^phox^ deficiency does not affect APP levels in *APP/PS1* mice. Representative Western blots showing APP expression in the hippocampus and the cortex of WT, *APP/PS1*, and *APP/PS1*-*Ncf1*^−/−^ mice aged 9 months (a) and 12 months (c). b, d Quantification of immunoreactivity of Western blots, normalized against GAPDH. **Figure S3.** p47^phox^ deficiency attenuates tau hyperphosphorylation in the cortex of *APP/PS1* mice and ICV-STZ mice. Representative Western blots showing tau phosphorylation at S199, T205, S396, and S404 in the cortex of WT, *APP/PS1*, *Ncf1*^*−/*−^, and *APP/PS1*-*Ncf1*^−/−^ mice aged 9 months (a) and 12 months (c), and of WT and *Ncf1*^−/−^ mice receiving ICV injection of STZ or saline at the age of 6 months (e). (b, d, f) Quantifications of the immunoreactivity of Western blots are shown. **Figure S4.** p47^phox^ deficiency attenuates tau aggregation and NTs in the hippocampus and the cortex of *APP/PS1* mice. (a) Representative immunofluorescence staining showing the decreased tau aggregation and NTs in *APP/PS1*-*Ncf1*^−/−^ mice aged 12 months, compared with age-matched *APP/PS1* mice. Quantification of the fluorescence of AT8 is shown. **Figure S5.** p47^phox^ deficiency attenuates NP tau in the hippocampus and the cortex of *APP/PS1* mice. Representative immunofluorescence staining of AT8 and thioflavin-S (Thio-S) showing the decreased NP tau in *APP/PS1*-*Ncf1*^−/−^ mice aged 12 months, compared with age-matched *APP/PS1* mice. **Figure S6.** The expression of NOX subunits (p47^phox^, p67^phox^, and NOX2/gp91^phox^) in *APP/PS1* mice. Representative Western blots showing p47^phox^, p67^phox^, and gp91^phox^ expression in the brain of WT and *APP/PS1* mice at the age of 9 months (a) and 12 months (c). (b, d) Quantifications of the immunoreactivity of Western bolts are shown. **Figure S7.** p47^phox^ deficiency inhibits the activation of astrocytes in *APP/PS1* mice. (a) Representative immunofluorescence staining showing the decreased expression of GFAP in *APP/PS1*-*Ncf1*^−/−^ mice aged 9 months and 12 months, compared with age-matched *APP/PS1* mice. (b) Quantification of the fluorescence of GFAP is shown. **Figure S8.** p47^phox^ deficiency inhibits the activation of microglia in *APP/PS1* mice. (a) Representative immunofluorescence staining showing the decreased expression of Iba1 in *APP/PS1*-*Ncf1*^−/−^ mice aged 12 months, compared with age-matched *APP/PS1* mice. (b) Quantification of the fluorescence of Iba1 is shown. **Figure S9.** p47^phox^ deficiency inhibits high glucose-induced activation of primary astrocytes. Primary cultures of astrocytes from WT and *Ncf1*^−/−^ newborn mice were treated with DMEM with or without high glucose (75 mM and 150 mM) for 24 h and 48 h. (a) Representative Western blots showing the expression of GFAP. (b, c) Quantification of the immunoreactivity of Western blots is shown.

## Data Availability

All datasets generated or analyzed during this study are available from the corresponding authors on reasonable request.

## Abbreviations

Aβ: Amyloid-β
AD: Alzheimer’s disease
APP: Amyloid precursor protein
Ara-C: Cytosine-β-D-arabinofuranoside
CM: Conditioned medium
DAPI: 4,6-Diamidino-2-phenylindole
DG: Dentate gyrus
DMEM: Dulbecco’s modified Eagle’s medium
GAPDH: Glyceraldehyde-3-phosphate dehydrogenase
GFAP: Glial fibrillary acidic protein
ICV: Intracerebroventricular
MWZ: Morris water maze
NADPH oxidase: Nicotinamide adenine dinucleotide phosphate oxidase
NFTs: Neurofibrillary tangles
NGS: Normal goat serum
OA: Okadaic acid
PMSF: Phenylmethanesulfonyl fluoride
ROS: Reactive oxygen species
SDS: Sodium dodecyl sulfate
STZ: Streptozotocin
Thio-S: Thioflavin-S
Trypsin-EDTA: Trypsin-ethylenediaminetetraacetic acid
