# Supplementary material for: p47phox deficiency improves cognitive impairment and attenuates tau hyperphosphorylation in mouse models of AD
Source: Alzheimers Res Ther. 2020 Nov 12;12:146. doi: 10.1186/s13195-020-00714-2 (PMC7659091; doi:10.1186/s13195-020-00714-2)
Supplement: Supplementary file 1 — Additional file 1: Figure S1. The effect of p47phox deficiency on the locomotor, spontaneous exploratory, and anxiety of APP/PS1 mice. The locomotion and spontaneous exploratory activity of mice aged 9 (a, b) and 12 (g, h) months was evaluated on Rotarod and open field tests, respectively. The anxiety of mice aged 9 months (c, d) and 12 months (i, j) was examined in open field and an elevated plus maze. Representative traces of a mouse’s movements during the open field test (e, k) and the elevated plus maze (f, l) are shown. Figure S2. p47phox deficiency does not affect APP levels in APP/PS1 mice. Representative Western blots showing APP expression in the hippocampus and the cortex of WT, APP/PS1, and APP/PS1-Ncf1−/− mice aged 9 months (a) and 12 months (c). b, d Quantification of immunoreactivity of Western blots, normalized against GAPDH. Figure S3. p47phox deficiency attenuates tau hyperphosphorylation in the cortex of APP/PS1 mice and ICV-STZ mice. Representative Western blots showing tau phosphorylation at S199, T205, S396, and S404 in the cortex of WT, APP/PS1, Ncf1−/−, and APP/PS1-Ncf1−/− mice aged 9 months (a) and 12 months (c), and of WT and Ncf1−/− mice receiving ICV injection of STZ or saline at the age of 6 months (e). (b, d, f) Quantifications of the immunoreactivity of Western blots are shown. Figure S4. p47phox deficiency attenuates tau aggregation and NTs in the hippocampus and the cortex of APP/PS1 mice. (a) Representative immunofluorescence staining showing the decreased tau aggregation and NTs in APP/PS1-Ncf1−/− mice aged 12 months, compared with age-matched APP/PS1 mice. Quantification of the fluorescence of AT8 is shown. Figure S5. p47phox deficiency attenuates NP tau in the hippocampus and the cortex of APP/PS1 mice. Representative immunofluorescence staining of AT8 and thioflavin-S (Thio-S) showing the decreased NP tau in APP/PS1-Ncf1−/− mice aged 12 months, compared with age-matched APP/PS1 mice. Figure S6. The expression of NOX subunits (p47 [file 13195_2020_714_MOESM1_ESM.pdf]

Additional file 1：

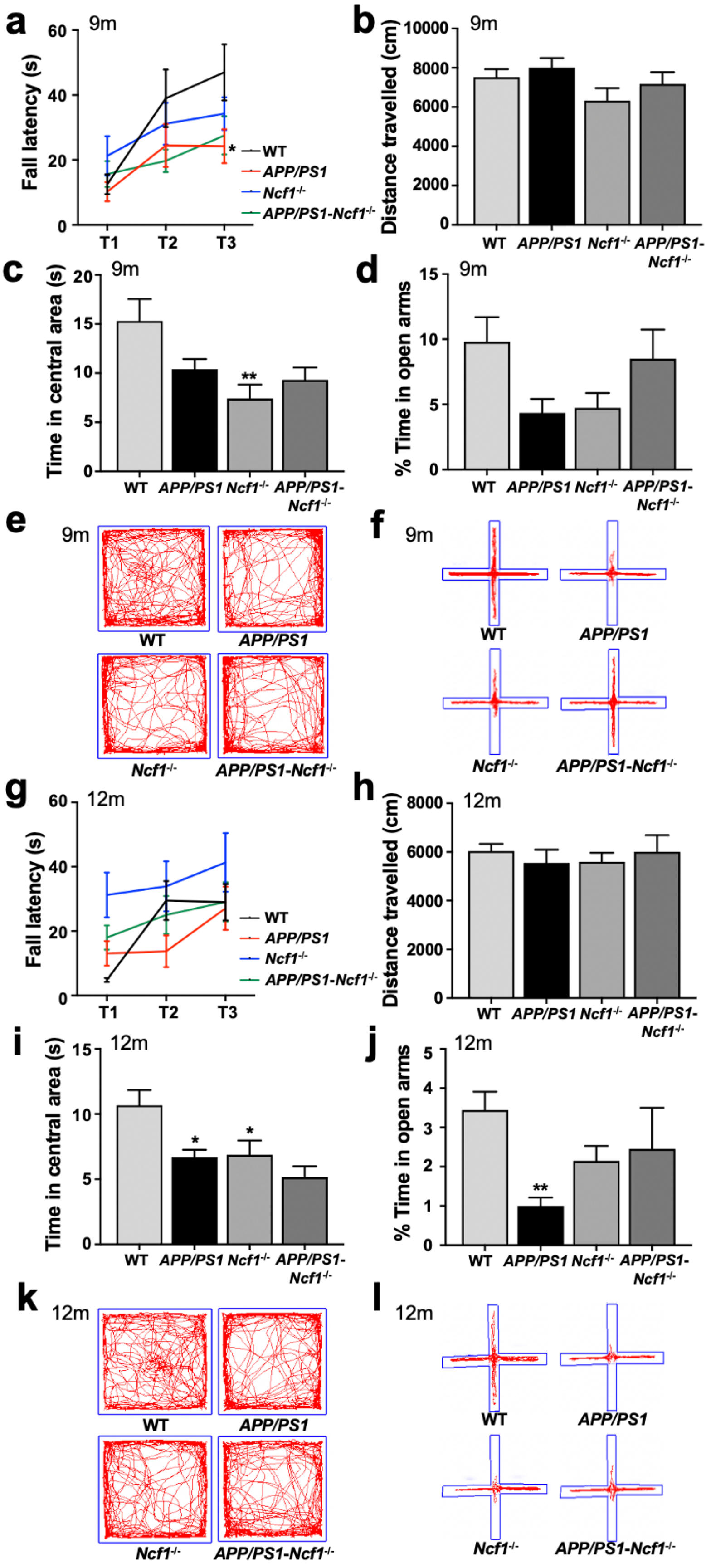

Fig. S1 The effect of p47^{phox} deficiency on the locomotor, spontaneous exploratory, and anxiety of *APP/PS1* mice. The locomotion of mice aged 9 months (**a**) and 12 months (**g**) was evaluated on Rotarod, and the fall latency was recorded. The spontaneous exploratory activity of mice aged 9 months (**b**) and 12 months (**h**) was tested in an open field, and the distance traveled during 15 min was recorded. The anxiety of mice aged 9 months (**c, d**) and 12 months (**i, j**) was examined in an open field and an elevated plus maze and the time spent in the center (**c, i**) and in the open arms (**d, j**) was recorded, respectively. Representative traces of a mouse's movements during the open field test (**e, k**) and the elevated plus maze (**f, l**) are shown. Two-way ANOVA: **a**, interaction F (6, 115) = 1.095 p = 0.3694, times F (2, 115) = 10.66 $p < 0.0001$, genotype F (3, 115) = 3.429 p = 0.0195; **g**, interaction F (6, 154) = 0.8967 p = 0.4990, times F (2, 154) = 5.104 p = 0.0071, genotype F (3, 154) = 4.458 p = 0.0049. One-way ANOVA: **b**, F (3, 39) = 1.515 p = 0.2258; **c**, F (3, 39) = 4.660 p = 0.0071; **d**, F (3, 37) = 2.264 p = 0.0972; **h**, F (3, 55) = 0.3642 p = 0.7791; **i**, F (3, 55) = 2.608 p = 0.0607; **j**, F (3, 47) = 4.385 p = 0.0084. Both male and female mice were used. Data are mean ± SEM, with 7-20 mice in each group. * $p < 0.05$, ** $p < 0.01$ compared with WT mice.

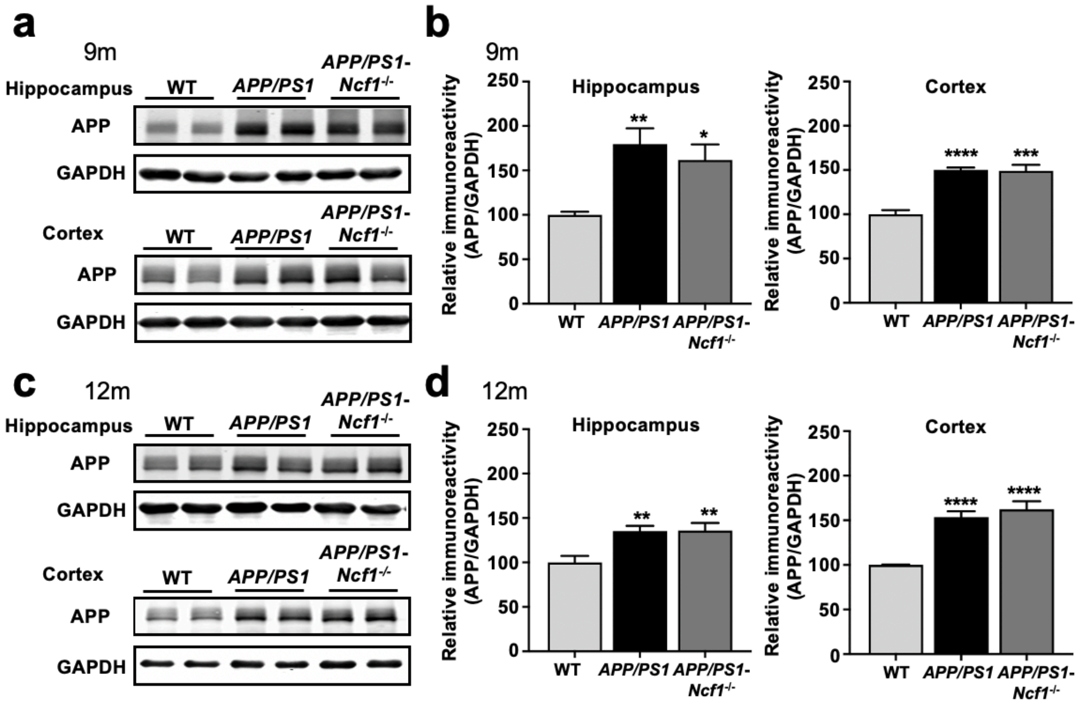

Fig. S2 p47^{phox} deficiency does not affect APP levels in *APP/PS1* mice. Representative Western blots showing APP expression in the hippocampus and the cortex of WT, *APP/PS1*, and *APP/PS1-Ncf1$^{-/-}$* mice aged 9 months (**a**) and 12 months (**c**). **b, d** Quantification of immunoreactivity of Western blots, normalized against GAPDH. One-way ANOVA: **b**, hippocampus F (2, 16) = 9.685 p = 0.0018, cortex F (2, 9) = 32.75 p < 0.0001; **d**, hippocampus F (2, 8) = 10.83 p = 0.0053, cortex F (2, 15) = 27.13 p < 0.0001. Both male and female mice were used. Data are mean ± SEM, with 4-5 mice in each group. ** p < 0.01, *** p < 0.001, **** p < 0.0001 compared with WT mice.

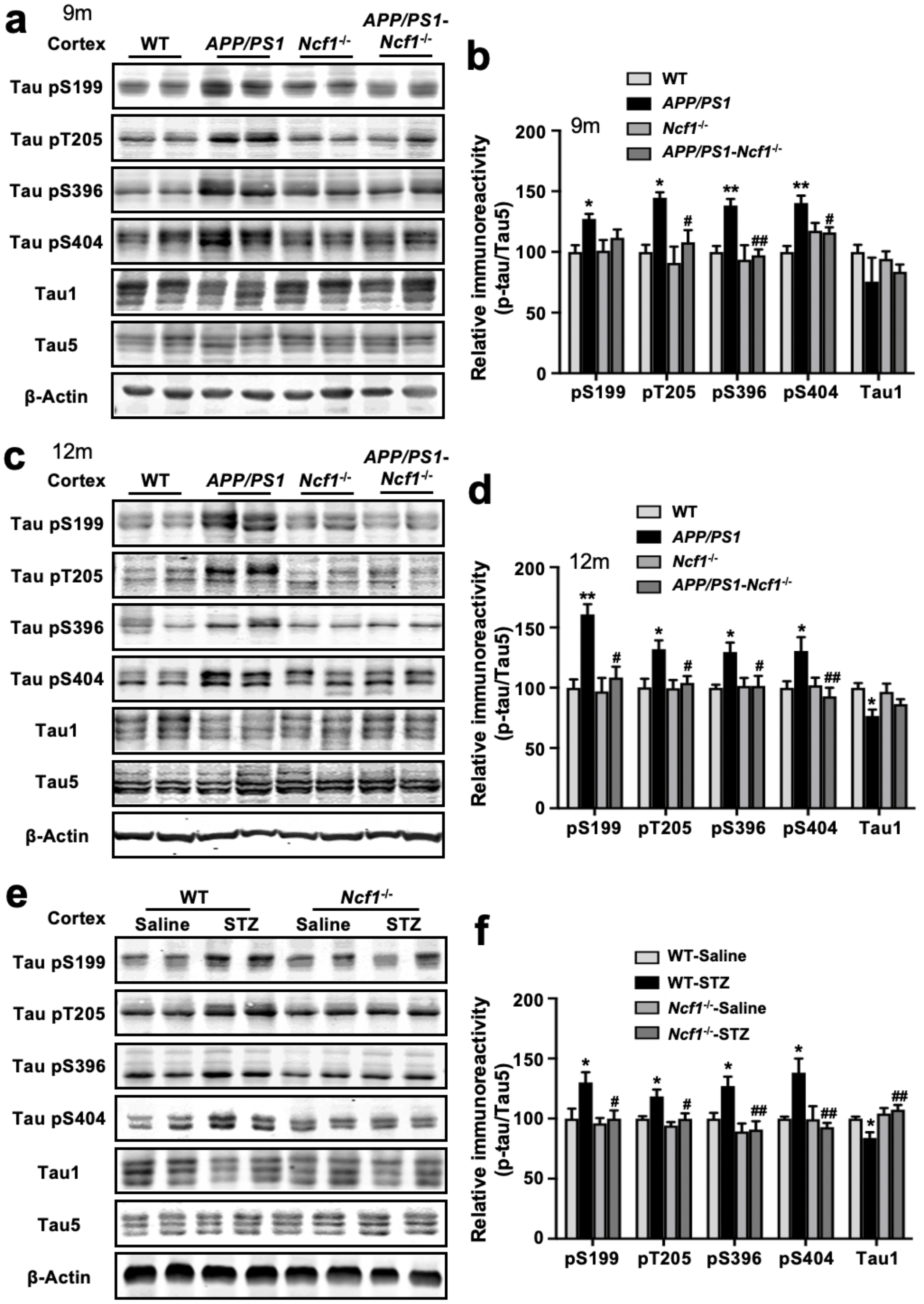

Fig. S3 p47^{phox} deficiency attenuates tau hyperphosphorylation in the cortex of *APP/PS1* mice and ICV-STZ mice. Representative Western blots showing tau phosphorylation at S199, T205, S396, and S404 in the cortex of WT, *APP/PS1*, *Ncf1*$^{-/-}$, and *APP/PS1-Ncf1*$^{-/-}$ mice aged 9 months (**a**) and 12 months (**c**). The levels of non-phosphorylated tau (Tau1) and total tau (Tau5) were also measured.

b, d Quantification of the immunoreactivity of Western blots, normalized against total tau. **e** Representative Western blots showing tau phosphorylation in the cortex of WT and $Ncf1^{-/-}$ mice receiving ICV injection of STZ or saline at the age of 6 months. **f** Quantification of the immunoreactivity of Western blots, normalized against total tau. One-way ANOVA: **b**, pS199 F (3, 12) = 3.066 p = 0.0691, pT205 F (3, 15) = 5.431 p = 0.0099, pS396 F (3, 17) = 6.790 p = 0.0033, pS404 F (3, 10) = 8.180 p = 0.0048, Tau1 F (3, 13) = 0.9222 p = 0.4575; **d**, pS199 F (3, 24) = 7.228 p = 0.0013, p205 F (3, 14) = 4.746 p = 0.0174, pS396 F (3, 33) = 3.241 p = 0.0344, pS404 F (3, 30) = 3.869 p = 0.0188, Tau1 F (3, 33) = 2.472 p = 0.0790; **f**, pS199 F (3, 23) = 5.026 p = 0.0080, pT205 F (3, 16) = 6.030 p = 0.0060; pS396 F (3, 20) = 7.146 p = 0.0019, pS404 F (3, 18) = 5.931 p = 0.0054, Tau1 F (3, 17) = 5.904 p = 0.0060. Both male and female mice were used. Data are mean ± SEM, with 4-6 mice in each group. * $p < 0.05$, ** $p < 0.01$ compared with WT or WT ICV-STZ mice; $^{\#}$ $p < 0.05$, $^{\#\#}$ $p < 0.01$ compared with *APP/PS1* or WT ICV-STZ mice.

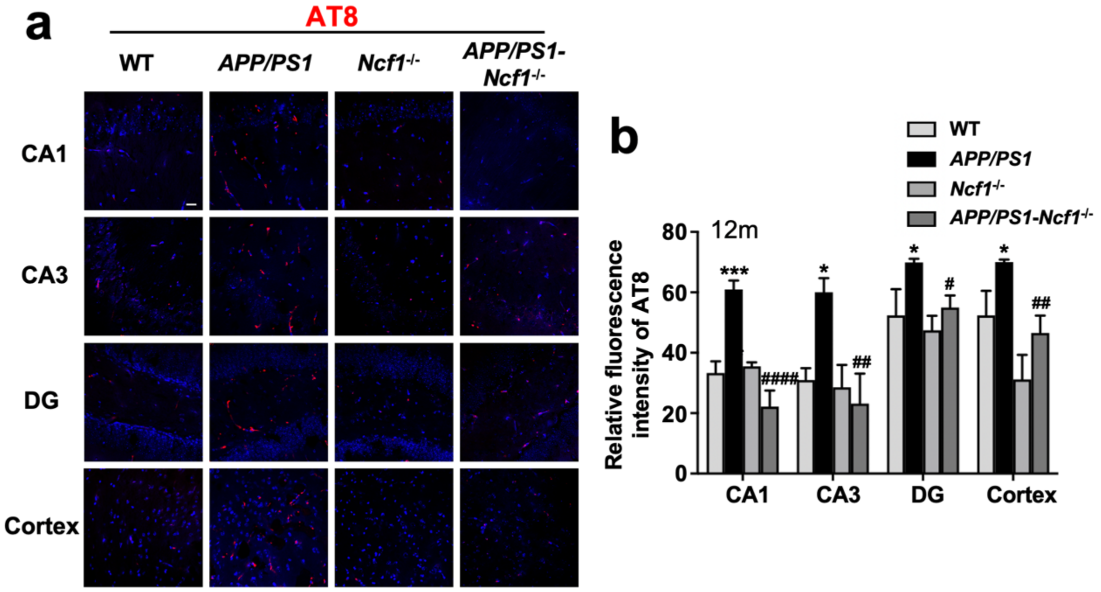

Fig. S4 p47phox deficiency attenuates tau aggregation and NTs in the hippocampus and the cortex of *APP/PS1* mice. **a** Serial sections of WT, *APP/PS1*, *Ncf1*$^{-/-}$, and *APP/PS1-Ncf1*$^{-/-}$ mice aged 12 months were stained for AT8 (*red fluorescence*) as described in *Materials and methods*. Nuclei were stained with DAPI (*blue fluorescence*). The scale bar in the upper left panel is 25 μm. **b** Quantification of the fluorescence of AT8 is shown. One-way ANOVA: CA1 F (3, 20) = 25.60 $p <$ 0.0001, CA3 F (3, 15) = 6.879 p = 0.0039, DG F (3, 20) = 5.943 p = 0.0045, Cortex F (3, 27) = 10.62 $p <$ 0.0001. Both male and female mice were used. Data are mean ± SEM, with three mice in each group. * $p < 0.05$, *** $p < 0.001$ compared with WT mice. $^{\#}$ $p < 0.05$, $^{\#\#}$ $p < 0.01$, $^{\#\#\#\#}$ $p <$ 0.0001 compared with *APP/PS1* mice.

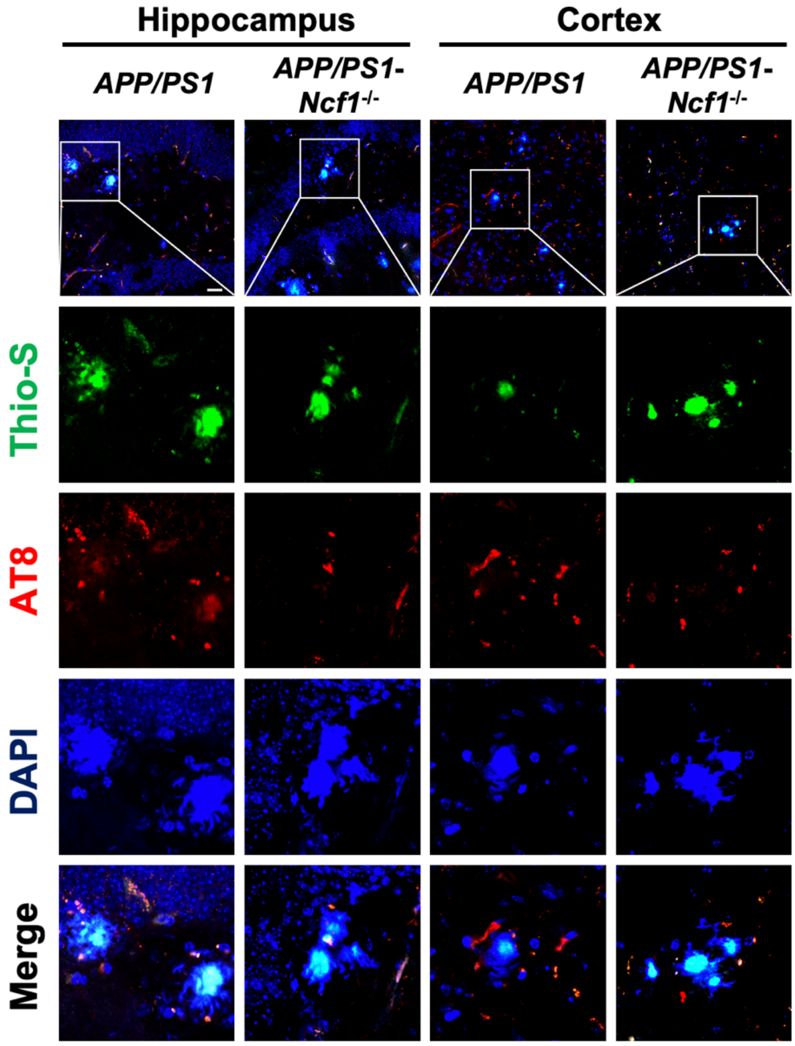

Fig. S5 p47^{phox} deficiency attenuates NP tau in the hippocampus and the cortex of *APP/PS1* mice. **a** Serial sections of *APP/PS1* and *APP/PS1-Ncf1*$^{-/-}$ mice aged 12 months were stained for AT8 (*red fluorescence*) and thioflavin-S (Thio-S, *green fluorescence*) as described in *Materials and methods*. Nuclei were stained with DAPI (*blue fluorescence*). The scale bar in the upper left panel is 25 µm. Selected areas are enlarged by nine times and shown as combined as well as individual fluorescence stains. Images shown are representative of multiple experiments (three mice in each group, both male and female mice were used).

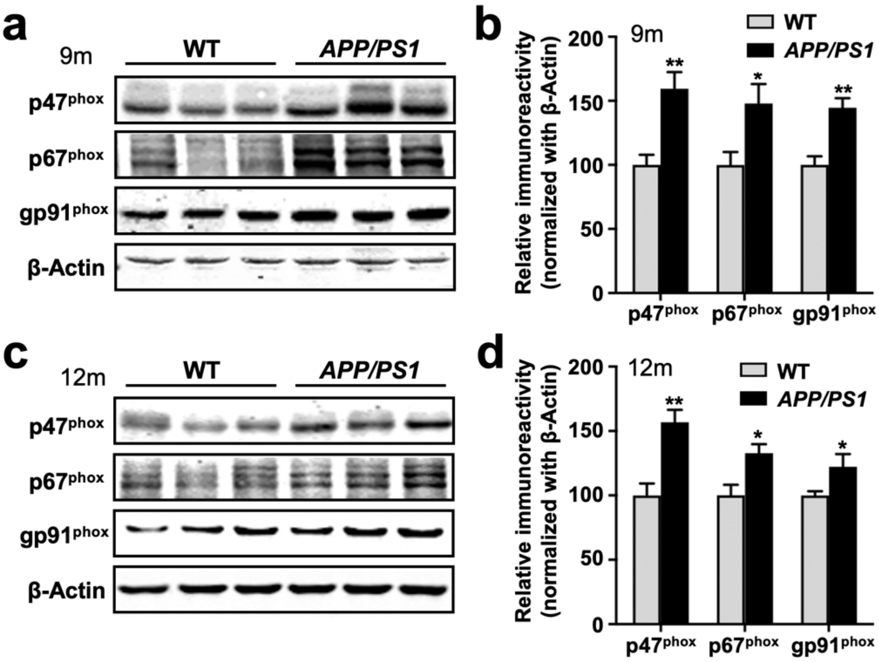

Fig. S6 The expression of NOX subunits ($p47^{phox}$, $p67^{phox}$, and NOX2/$gp91^{phox}$) in *APP/PS1* mice. Representative Western blots showing $p47^{phox}$, $p67^{phox}$, and $gp91^{phox}$ expression in the brain of WT and *APP/PS1* mice at the age of 9 months (**a**) and 12 months (**c**). **b, d** Quantification of the immunoreactivity of Western bolts, normalized against β-Actin. Both male and female mice were used. Data are mean ± SEM, with 5-6 mice in each group. * $p < 0.05$, ** $p < 0.01$ compared with WT mice.

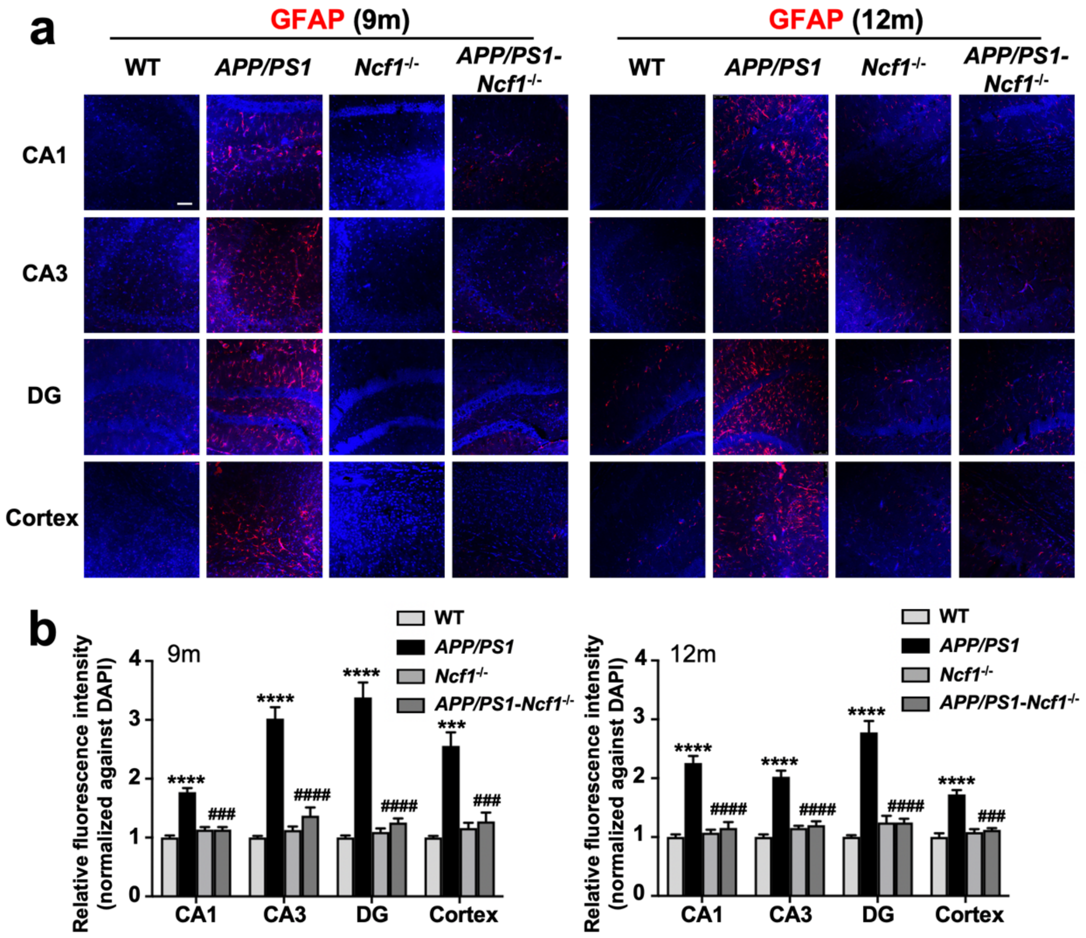

Fig. S7 p47[phox] deficiency inhibits the activation of astrocytes in *APP/PS1* mice. **a** Serial sections of WT, *APP/PS1*, *Ncf1*$^{-/-}$, and *APP/PS1-Ncf1*$^{-/-}$ mice aged 9 months and 12 months were stained for GFAP (*red fluorescence*) as described in *Materials and methods*. Nuclei were stained with DAPI (*blue fluorescence*). The scale bar in the upper left panel is 75 μm. **b** Quantification of the fluorescence of GFAP is shown. One-way ANOVA: **b**, 9m, CA1 F (3, 9) = 46.90 $p < 0.0001$, CA3 F (3, 8) = 53.46 $p < 0.0001$, DG F (3, 9) = 77.97 $p < 0.0001$, Cortex F (3, 8) = 27.39 $p = 0.0001$; 12m, CA1 F (3, 8) = 48.00 $p < 0.0001$, CA3 F (3, 8) = 47.20 $p < 0.0001$, DG F (3, 8) = 48.53 $p < 0.0001$, Cortex F (3, 8) = 34.86 $p < 0.0001$. Both male and female mice were used. Data are mean ± SEM, with three mice in each group. *** $p < 0.001$, **** $p < 0.0001$ compared with WT mice. $^{###}$ $p < 0.001$, $^{####}$ $p < 0.0001$ compared with *APP/PS1* mice.

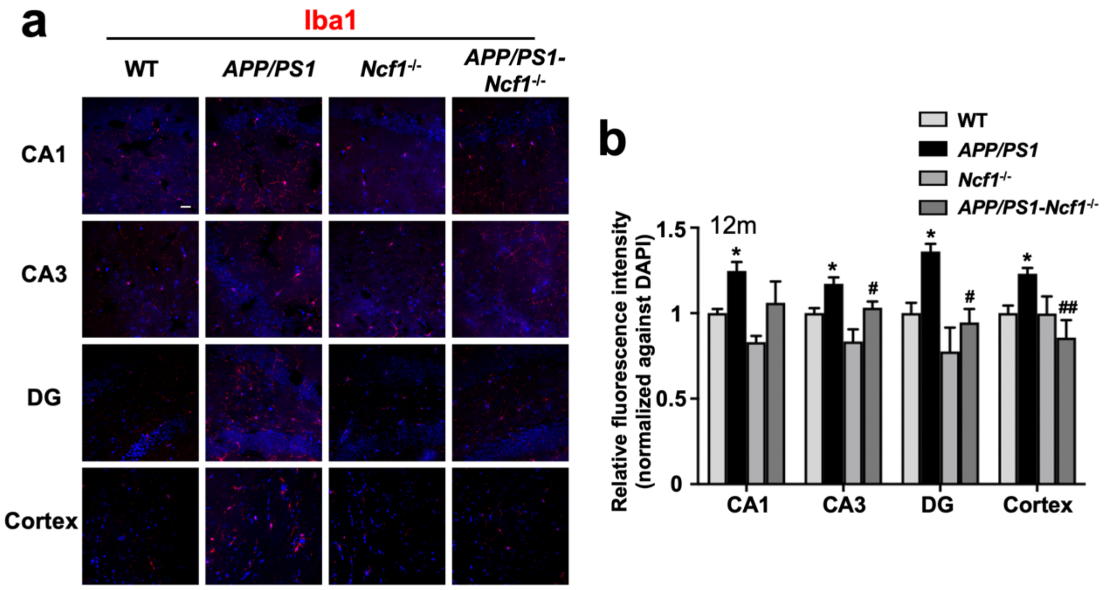

Fig. S8 p47^{phox} deficiency inhibits the activation of microglia in *APP/PS1* mice. **a** Serial sections of WT, *APP/PS1*, *Ncf1*$^{-/-}$, and *APP/PS1-Ncf1*$^{-/-}$ mice aged 12 months were stained for Iba1 (*red fluorescence*) as described in *Materials and methods*. Nuclei were stained with DAPI (*blue fluorescence*). The scale bar in the upper left panel is 25 µm. **b** Quantification of the fluorescence of Iba1 is shown. One-way ANOVA: CA1 F (3, 16) = 8.337 p = 0.0014, CA3 F (3, 13) = 9.004 p = 0.0017, DG F (3, 14) = 7.025 p = 0.0041, Cortex F (3, 18) = 5.417 p = 0.0078. Both male and female mice were used. Data are mean ± SEM, with three mice in each group. * $p < 0.05$, ** $p < 0.01$ compared with WT mice. # $p < 0.05$ compared with *APP/PS1* mice.

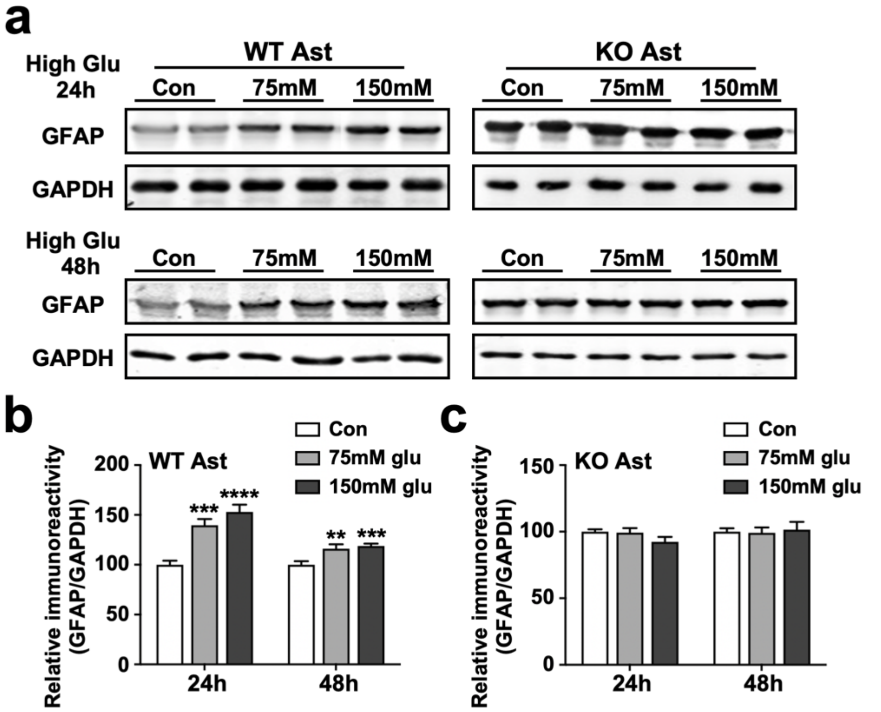

Fig. S9 p47[phox] deficiency inhibits high glucose-induced activation of primary astrocytes. Primary cultures of astrocytes from WT and *Ncf1*$^{-/-}$ newborn mice were treated with DMEM with or without high glucose (75 mM and 150 mM) for 24 h and 48 h. **a** Representative Western blots showing the expression of GFAP. **b, c** Quantification of the immunoreactivity of Western blots, normalized against GAPDH. One-way ANOVA: **b**, 24h F (2, 18) = 20.66 $p < 0.0001$, 48h F (2, 18) = 10.26 $p = 0.0011$; **c**, 24h F (2, 20) = 1.808 $p = 0.1897$, 48h F (2, 21) = 0.08514 $p = 0.9187$. Data are mean ± SEM from three separate experiments, each in duplicate or triplicate. ** $p < 0.01$, *** $p < 0.001$, **** $p < 0.0001$ compared with medium without high glucose.
